# Recent Advances in the Applications of Biomaterials in Ovarian Cancer

**DOI:** 10.3390/biomimetics10110768

**Published:** 2025-11-12

**Authors:** A M U B Mahfuz, Amol V. Janorkar, Rodney P. Rocconi, Yuanyuan Duan

**Affiliations:** 1 Department of Biomedical Materials Science, University of Mississippi Medical Center, Jackson, MS 39216, USA; amahfuz@umc.edu (A.M.U.B.M.); ajanorkar@umc.edu (A.V.J.); 2 Cancer Center and Research Institute, University of Mississippi Medical Center, Jackson, MS 39216, USA; rocconi@umc.edu

**Keywords:** biomaterial, nanomaterial, ovarian cancer, drug delivery, tumor diagnosis

## Abstract

Among the gynecological cancers, ovarian cancer is the most fatal. Despite advancements in modern medicine, the survival rate is abysmally low among ovarian cancer patients. Ovarian cancer poses several unique challenges, like late diagnosis due to the initial vagueness of the symptoms and lack of effective screening protocols. Recently, biomaterials have been explored and utilized extensively for the diagnosis, treatment, and screening of ovarian malignancies. Biomaterials can help bypass the obstacles of traditional chemotherapy and enhance imaging capabilities. They are also indispensable for next-generation biosensors and tumor organoids. Biomaterials inspired by biomimetic strategies that replicate the structural, chemical, and functional properties of natural biological systems have proven to have better functionalities. While numerous review articles have examined biomaterials in oncology, there is a lack of reviews dedicated specifically to their applications in ovarian cancer. This review aims to address this critical gap by providing the first comprehensive overview of the current biomaterial research on ovarian cancer and highlighting key challenges, opportunities, and future directions in this evolving interdisciplinary field.

## 1. Introduction

In today’s world, ovarian cancer is responsible for the highest mortality among women suffering from gynecological cancers. In 2022 alone, 324,398 new ovarian cancer patients were diagnosed, and 206,839 women died of ovarian cancer worldwide [1]. In spite of the recent advances achieved in modern medicine, the 5-year survival rate for this cancer is dismally low, particularly among cases diagnosed at advanced stages (≤20% for stage III and IV). The asymptomatic, mildly symptomatic, or vaguely symptomatic nature of the early-stage disease and the absence of sensitive screening methods are responsible for late diagnosis and subsequent high mortality. Almost 80% of patients reach stage III or IV when they are diagnosed [2]. Furthermore, the heterogeneity of ovarian cancer at the molecular, histological, and anatomical levels presents significant challenges in designing universally effective treatments [3]. Standard therapeutic approaches include cytoreduction surgeries and adjuvant platinum-based chemotherapy. Although they initially offer good response rates, recurrence is common and often accompanied by chemoresistance [4]. This necessitates newer therapies and approaches with superior efficacy for early diagnosis and minimizing off-target effects.

Ovarian cancer develops when cells in the ovaries or fallopian tubes undergo malignant transformation and begin to grow uncontrollably. This disease typically starts with epithelial cells (about 90% of cases), stromal cells, or germ cells. According to the current WHO classification of tumors of the female genital organs [5], there are five principal histotypes of primary epithelial ovarian carcinoma: high-grade serous, low-grade serous, endometrioid, clear cell, and mucinous. Serous carcinomas (both high- and low-grade) often originate in the epithelium of the fallopian tube. Cancerous cells from the Fallopian tube can spill on the ovary and cause disease progression [6,7].

Risk factors include genetic mutations (BRCA1/BRCA2, Lynch syndrome), family history, age, nulliparity, endometriosis, and hormone replacement therapy. The cancer often spreads by shedding cells into the peritoneal cavity, where they implant on different abdominal organs. Early-stage ovarian cancer typically produces nonspecific symptoms. These include abdominal bloating or distension, pain in the pelvis or abdomen, feeling full quickly during eating, and frequent urge to urinate. Some women notice increased abdominal girth due to ascites or a palpable abdominal mass [7,8,9].

High-grade serous ovarian carcinoma (HGSOC) is the most aggressive and prevalent form of epithelial ovarian cancer. This alone accounts for approximately 70–80% of cases [10]. Its distinguished feature is TP53 mutation in nearly all cases and frequent genomic instability. HGSOC typically presents at an advanced stage with widespread intraperitoneal dissemination and malignant ascites. In contrast, low-grade serous carcinoma exhibits KRAS, BRAF, or ERBB2 mutations and progresses slowly from serous borderline tumors. Endometrioid and clear cell carcinomas are commonly associated with endometriosis and harbor ARID1A, PTEN, and PIK3CA mutations. Mucinous carcinomas are often unilateral and large. They are linked to KRAS mutations and may resemble gastrointestinal tumors. These biological and molecular distinctions have important therapeutic implications. HGSOC tends to respond initially to platinum-based chemotherapy but relapses frequently. Non-serous histotypes may be less chemosensitive but more amenable to targeted or hormonal therapies [11,12,13,14].

Biomaterials are rising tools in ovarian cancer research and treatment. A material can be considered a biomaterial if it comes in contact with a biological system for evaluation, treatment, augmentation, or replacement of any body part [15]. Over the last few decades, biomaterials have revolutionized various aspects of medicine—artificial heart valves, prostheses, implants, regenerative therapies, drug delivery, and imaging, to name a few. Their tunable properties, biocompatibility, and scopes for functional modification make them especially attractive for cancer applications [16]. From diagnosis to treatment, biomaterials can be utilized at every step of ovarian cancer care. Nanoparticles (NPs), hydrogels, liposomes, polymeric scaffolds, and other biomaterial-based platforms hold promising potential for better diagnosis, targeted drug delivery, precision imaging, and even in vitro tumor modeling. They offer ways to bypass biological barriers, reduce systemic toxicity, and directly deliver cargo to the target sites, thereby improving the therapeutic index [17].

Biomimetics or biomimicry is the design and fabrication of a product where its structure, mechanisms, or functions replicate natural entities. In the context of ovarian cancer research, the integration of biomimetic design principles in biomaterials can lead to breakthroughs in diagnosis and therapy. By mimicking the extracellular matrix, cellular membranes, or tumor microenvironmental cues, biomaterials can achieve selective targeting, controlled drug release, and immune modulation that closely resemble natural processes. Examples include cancer cell membrane-coated nanoparticles to bypass immune clearance, folate receptor-targeted liposomes inspired by ligand–receptor specificity, and matrix-mimicking scaffolds that replicate the properties of ovarian stroma. These biomimetic systems can intelligently interact with the surrounding biological environment instead of merely coexisting.

The applications of biomaterials in ovarian cancer are summarized in this narrative review article. We conducted a literature search in the SCOPUS database using the following keywords: “ovarian cancer AND (liposome OR niosome OR polymersome OR dendrimer OR micelle OR fullerene OR nanomaterial OR nanostructure * OR nanoparticle OR nanocarrier OR nanocapsule OR nanoemulsion OR nanogel OR nanotube OR hydrogel)”. The time span was set from 2020 to the current day. Only the research articles and the articles published in the English language were considered. A total of 1070 articles were retrieved, and those demonstrating broader application potential were selected for further analysis. Some articles outside of this list but included in PubMed or Web of Science were also included for the sake of completeness. In this work, recent advancements in this field have been highlighted to keep the researchers working at the intersection of biomaterials and ovarian cancer updated. By focusing on current challenges and future perspectives, research gaps have been identified, and future directions have been discussed. Through integrating knowledge across materials science, oncology, and drug delivery, this review article seeks to provide a comprehensive understanding of how biomaterials can redefine the clinical management of ovarian cancer.

## 2. Applications of Biomaterials in Ovarian Cancer Diagnosis

### 2.1. Nanomaterials for Enhanced Imaging

Circulating tumor cells are responsible for metastasis to organs distant from the primary site. CXCR4 is a G-protein-coupled receptor. Overexpression of CXCR4 in many solid tumors is the culprit behind their progression and metastasis. CXCL12 is a chemokine that preferentially binds to CXCR4. Portella et al. developed a CXCL12-loaded hyaluronic acid-based hydrogel for the capture, characterization, and enumeration of circulating ovarian cancer cells. They conducted a clinical trial on 48 cancer patients with metastasis to assess the efficacy of their platform. This specialized hydrogel was successful in capturing cancer cells that were visualized under a fluorescent microscope. This platform could be a potential tool for early metastasis detection and therapeutic monitoring [18]. Beyond cell capture, nanomaterials are being leveraged to enhance both diagnostic imaging and therapeutic delivery. Wu et al. engineered a system from manganese-doped mesoporous silica nanoparticles for delivering immunotherapy, chemotherapy, and imaging agents to ovarian cancer tissue. This system trapped anti-programmed cell death-ligand 1 antibody (aPD-L1) and paclitaxel (PTX) within a polydopamine capsule. The capsule was designed to be pH- and glutathione (GSH)-sensitive, which allowed it to degrade in the low acidic and reductive tumor microenvironment. The degradation released Mn^2+^, a contrast agent for magnetic resonance imaging (MRI), as well as PTX, which directly caused death to tumor cells. Another content was aPD-L1, which induced tumor cell killing by immunogenic reaction [19].

Fluorescent nanoparticles can detect the distribution of ovarian cancer cells during the preoperative, intraoperative, and postoperative stages. Near-infrared (NIR) aggregation-mediated emission luminogens (AIEgens) offer real-time, sensitive imaging by avoiding aggregation-caused quenching (ACQ). β-Galactosidase (β-Gal) is highly expressed in ovarian carcinoma. It has been challenging to image β-Gal in vivo using the existing visible range AIEgens. Xu et al. designed an NIR-AIEgen (named QM-TPA-Gal) for the imaging of β-Gal. Once they enter ovarian cancer cells, β-Gal hydrolyzes the hydrophilic, non-fluorescent QM-TPA-Gal to hydrophobic QM-TPA-OH. This conversion causes the molecules to aggregate, which activates NIR fluorescence (peak at ∼675 nm). In vitro experiments demonstrated that QM-TPA-Gal is highly sensitive and selective towards β-Gal (0.21 U/mL is the detection limit). This NIR AIEgen can be an important player in the early diagnosis of β-Gal-associated tumors [20].

Folate receptor (FR) is overexpressed in many cancers. Ovarian and cervical carcinoma are prominent among them. Targeting overexpressed cell surface markers has also proven effective in enhancing diagnostic precision and therapeutic delivery. Quindoza and colleagues focused on modifying luminescent europium-doped hydroxyapatite (EuHAp) nanocrystals with folic acid (FA) to improve diagnostic accuracy. They synthesized EuHAp nanocrystals using a hydrothermal method, and then FA was grafted on the nanocrystals with the help of (3-Aminopropyl)triethoxysilane (APTES) linkages. Confocal microscopic images confirmed the successful targeting and subsequent internalization of these nanocrystals in HeLa cells. Fluorescence microscopy showed a 120-fold rise in signal due to enhanced uptake of the modified EuHAp particles by HeLa cells. These nanocrystals can be utilized to detect ovarian and breast cancer cells overexpressing FA receptors [21].

Dai and coworkers loaded tri(poly(phenylphosphine)), an aggregation-induced emission (AIE) molecule, on hollow mesoporous silica nanoparticles (HMSN). They cloaked these nanoparticles with cancer-associated fibroblasts’ (CAF) membrane. The negative potential of this membrane increases the circulation time of these nanoparticles. Also, fibronectin present on the CAF membrane binds to integrin α-5, a protein overexpressed on the ovarian cancer cell membrane. The signal-to-noise (S/N) ratio of HMSN-CAF nanoparticles was 8.6:1. These nanoparticles can be utilized for monitoring at preoperative, intraoperative, and postoperative stages of ovarian cancer [22].

**Figure 1:**
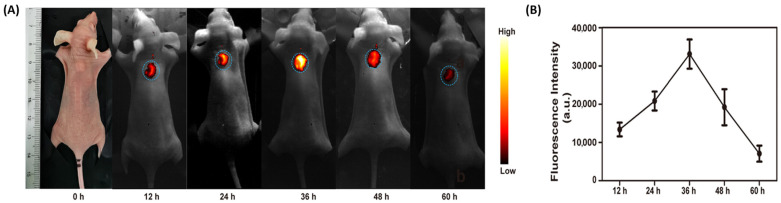
(**A**) NIR-II fluorescence imaging of subcutaneous tumors over a period of 60 h after injecting the NPs prepared by Pu et al., 2023 [24]. (**B**) Maximum intensity was observed after 36 h. So, the NPs should be injected 36 h before surgery for the best visualization. Reproduced from Pu et al., 2023, used under a Creative Commons CC-BY-NC-ND 4.0 license [24].

Li and coworkers engineered a fluorescent selenium (Se) nanoparticle for precise ovarian cancer diagnosis. This nanoprobe is functionalized with RGD peptides and S2.2 aptamer (targets MUC1) for high-affinity recognition of malignant ovarian tissues. Se@RGD/S2.2 facilitates rapid uptake and noninvasive visualization of microinvasive lesions. This approach helps determine the stage of ovarian cancer and potentially will enhance patient outcomes [23]. Using benzobisthiadiazole, a near-infrared-II fluorescent (NIR-II) dye, and DSPE-PEG, Pu and coworkers created nanoparticles of approximately 80 nm diameter that demonstrated high lymphophilicity as well as extended circulation time. Strong NIR-II emission (~1060 nm) by these nanoparticles made it possible to image orthotopic (a tumor model where cancer cells are implanted into the corresponding organ of an animal) ovarian tumors with a high S/N ratio (13.4:1) (Figure 1A). These nanoparticles detected tumor-regional lymph nodes and sub-millimeter peritoneal metastases (>5 S/N ratio) in advanced-stage ovarian cancer models up to 36 h post-administration (Figure 1B). Image-guided surgery using these nanoparticles facilitated precise tumor resection and accurate surgical staging. This underscores their potential in the progress of ovarian cancer treatment [24]. The superior performance of NIR-II probes becomes evident when their S/N ratios are compared with others. While HMSN-CAF particles achieved an S/N ratio of 8.6:1, NIR-II nanoparticles reached an S/N ratio of 13.4:1 and maintained an S/N ratio of >5:1 even for sub-millimeter metastases. This 36 h detection window far exceeds typical AIEgen imaging timeframes, demonstrating the critical advantage of longer wavelengths for clinical translation in surgical guidance.

**Figure 2:**
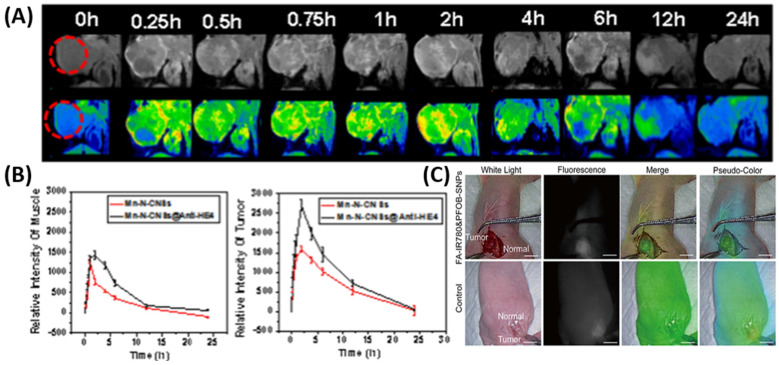
(**A**) (**Top**) T1-weighted MRI and (**bottom**) corresponding pseudo-color fluorescence images after injecting the bi-modal imaging agent containing anti-HE4 antibody. The red circle indicates the tumor. (**B**) Intensity of signal in the muscles (**left**) and in the tumors (**right**). Reproduced from Han et al., 2020, used under a Creative Commons CC BY 4.0 license [36]. (**C**) Fluorescence imaging at 72 h post-intraperitoneal injection. The bar indicates 1 cm. Reproduced from Song et al., 2022, used under a Creative Commons CC BY-NC 3.0 license [26].

Kumar et al. successfully conjugated luteinizing hormone-releasing hormone (LHRH) on the surface of Au nanoparticles. Spectral photon-counting CT imaging confirmed the uptake of these nanoparticles by ovarian cancer cells [25]. In a similar attempt, Song et al. developed nanoparticles from PLGA and PEG and used folate to target ovarian cancer cells. Perfluorooctyl bromide was used to enhance the stability of these nanoparticles. These nanoparticles were loaded with IR780 dye for fluorescence imaging and were successful in identifying even micro-sized tumors in subcutaneous and peritoneal xenograft tumor models of ovarian cancer. They can, thus, ensure greater efficacy during tumor resection. This imaging modality holds promise for improving surgical precision and resection completeness (Figure 2C) [26]. Urokinase plasminogen activator receptor (uPAR), a protein overexpressed in ovarian cancer cell membranes, serves as an appealing molecular target for diagnostic imaging. The somatomedin B domain (SMB) and growth factor domain (GFD) have affinities toward uPAR. Shahdeo and colleagues functionalized chitosan-coated iron oxide nanoparticles with GFD and SMB to precisely locate ovarian cancer cells [27].

Vankayala et al. repurposed genome-depleted brome mosaic virus (which infects plants) as nanocarriers. They removed the genome from the virus and loaded NIR brominated cyanine dye (BrCy106-NHS) on the virus particles. These nanoparticles were surface-modified with anti-HER2 receptor antibodies for effective targeting of ovarian cancer cells. Intraperitoneal ovarian tumors in mice showed effective uptake of these genome-depleted viral particles and a high S/N ratio [28]. Burns and colleagues engineered nanoparticles from red blood cells (RBC) and encapsulated indocyanine green within the nanoparticles (nRBCs-ICG) to build a near-infrared imaging probe. This was used for superior visualization during ovarian tumor resection surgery. Spatially modulated illumination at frequencies of 0–0.5 mm^−1^ enabled high-contrast fluorescence imaging of intraperitoneal ovarian tumors in mice. Mouse studies further disclosed preferential accumulation at intraperitoneal tumor sites 24 h after injection compared to other organs [29].

Hada and coworkers stabilized gold nanoclusters with the help of bovine serum albumin and functionalized them with FA for targeted imaging in ovarian malignancies. On average, these nanoparticles were 2–3 nm in size and demonstrated stable emission in the first biological window. Fluorescence lifetime imaging microscopy demonstrated their enhanced uptake and imaging performance [30]. Similarly, focusing on gold nanoparticles, Kumar et al. converted the bromo terminal of 11-bromoundecanoic acid (UA) into a thiol (-SH) functionality and conjugated FA and hydrazinonicotinic acid (HYNIC) to obtain FA-UA-SH and HYNIC-UA-SH, respectively. Reactions of these chemical entities with chloroauric acid yielded FA and HYNIC-functionalized gold nanoparticles. They then radiolabeled these nanoparticles with ^99m^Tc. However, in the mouse model of folate receptor-positive tumor, uptake of these particles was 1.39 ± 0.18% ID/g in the tumor, whereas in renal tissues, it was 5.48 ± 0.72% ID/g after 3 h of injection. This suggests that this approach needs to be modified for better targeting and reducing nephrotoxicity [31].

Multimodal platforms of imaging utilize different complementary approaches to overcome the limitations of a single method. Zhang and coworkers modified PLGA nanoparticles with cyclic RGD peptide and loaded perfluorohexane and Fe_3_O_4_ for dual-mode imaging (ultrasonogram and MRI, respectively). BIRC5 is responsible for resistance to platinum-based chemotherapy. They also loaded small interfering RNA targeting the *BIRC5* gene and prodrug platinum (IV) on those nanoparticles to overcome resistance in ovarian cancer cells [32]. Semiconductor nanoplatelets, characterized by their ultrathin morphology comprising only a few atomic layers, offer unique optical properties for bioimaging. Polymeric nanocarriers from poly(ethylene oxide) and poly(propylene oxide) block copolymers were prepared by Nawrot et al. to encapsulate CdSe semiconductor nanoplatelets for two-photon microscopy. These nanocarriers can enter MDAH-2774 ovarian cancer cells. 2.2 × 10^7^ GM (Goeppert-Mayer units) two-photon brightness was achieved at 870 nm using these nanoparticles. Although in vivo validation is pending, these nanoparticles showed almost identical results within the physiological pH and temperature ranges [33].

Kantamneni and colleagues synthesized albumin-based nanoprobes doped with rare-earth elements (holmium, erbium, and thulium) for short-wave infrared (SWIR) imaging applications. They further modified the nanoprobes with daidzein, folic acid, and AMD3100 to target caveolin-1, folate receptor alpha, and CXCR4, respectively. These nanoprobes emit light in the 900–1700 nm wavelength region (SWIR region). Light absorption and scattering in the SWIR region are relatively less than in the visible and NIR range. In vitro and in vivo experiments in ovarian (SKOV3) and breast (MCF-7 and 4175) cancer models demonstrated that these nanoprobes accumulated more in the cells that overexpressed the targeted receptors than in cells that did not [34]. Asgari and colleagues first synthesized carbon dot (CD) conjugated superparamagnetic iron oxide nanoparticles (SPIONs). They later attached the TOV6 aptamer to the SPIONs for targeting ovarian cancer cells. Because of the presence of both carbon dots and SPION, these combined nanoparticles were found to act both as a fluorescence probe and an MRI contrast agent [35]. Human epididymis protein 4 (HE4) is a biomarker of ovarian cancer. Similar to Asgari et al., Han et al. developed an MRI/fluorescence bi-modal imaging agent by conjugating the anti-HE4 antibody to carbon nanosheets doped with manganese and nitrogen (Figure 2A,B) [36].

Nanomaterial-based approaches can revolutionize ovarian cancer imaging by enabling multimodal, high-sensitivity, and targeted visualization of tumors. AIEgens stand out for their aggregation-induced emission, whereas NIR-II and SWIR probes excel in deep-tissue penetration and minimal background interference. Metal-based and carbon-derived nanomaterials can contribute additional MRI contrast and photo-stability. Biomimetic coatings like CAF membranes or RBC-derived vesicles improve circulation and biocompatibility. Future developments in this area should prioritize utilizing synergistic material properties, easy synthesis, precise delivery, long fluorescence time, and safe excretion from the body.

### 2.2. Biosensors for Detecting Ovarian Cancer Markers

Cancer antigen 125 (CA-125) acts as both a diagnostic as well as a prognostic biomarker of ovarian cancer. Wu et al. constructed a ratiometric electrochemical immunosensor for detecting CA-125. They used three-dimensional reduced graphene oxide and multi-walled carbon nanotube with a -COOH group (MWCNT-COOH) composites to capture CA-125. The presence of MWCNTs prevented the aggregation of graphene sheets, and rGO/MWCNT composites formed a 3D structure of higher conductivity and surface area. Because of its higher surface area, this structure could entrap more thionine, an electrochemical probe, and its higher conductivity helped amplify the signal from thionine. Ferrocene-carboxylic acid has good anti-interference properties and biocompatibility. UIO-66-ferrocenecarboxylic acid also sent a strong signal after binding to CA-125. Thus, two distinct signals from this immunosensor confirmed the presence of CA-125. This sensor works optimally at pH 6 [37]. Gharehaghaji et al. invented an electrochemical immunosensor that is capable of detecting trace amounts of CA-125. The working potential range of this sensor is −0.2 to 0.6 V, whereas the limit of detection (LOD) is 0.075 nU/mL. They polished a glassy carbon electrode, ultrasonically rinsed it, and modified it with MXene-graphene quantum dots. Then, Au nanoparticles were electrochemically deposited on it. This modification improved the electrode’s conductivity and surface area. Finally, streptavidin and CA-125 antibody were added sequentially on its surface to create the immunosensor [38]. Er et al. synthesized organohydrogel structures from glycerol, glutaraldehyde, agar, and onion oil. They prepared an electrochemical sensor for detecting serum CA-125 using the organohydrogel, and the LOD value of this sensor is 0.805 μU/mL [39].

These initial CA-125 detection strategies reveal a common design principle: nanomaterial composites can significantly enhance both the surface area for antibody immobilization and the electrical conductivity for signal amplification. While Wu’s ratiometric approach offers the advantage of dual-signal confirmation to reduce false positives, Gharehaghaji’s MXene-based sensor achieves superior sensitivity with an impressively low LOD. Table 1 gives an idea of the different electrochemical sensors developed for detecting CA-125.

**Table 1:** Electrochemical sensors for detecting CA-125 [Adapted from Er. et al., 2024 [39], used under a Creative Commons CC BY 4.0 license].

Sensor	Concentration Range	LOD	References
MPA/AuNPs@SiO2/QD/mAb	0–0.1 U/mL	0.0016 U/mL	[40]
Ag NPs-GQDs/Ab/BSA/Ag	0.01 U/mL	0.01–400 U/mL	[41]
Au-PB-PtNP-PANI hydrogel/GCE	0.01–5000 U/mL	4.4 mU/mL	[42]
Ab2–Ag–Ab1/Au-VBG/BDD/Ta	0.09 mU/mL	0.5–100 U/mL	[43]
BSA/Ab/Au NPs/Cys A/ERGO-P(DA)-GCE	0.1 U/mL	0.1–400 U/mL	[44]
FA@H-PANI@CS-HCl	0.25 pg/mL	0.001–25 ng/mL	[45]
CuO nanoflakes	0.77–500 IU/mL	0.77 IU/mL	[46]
MOF-808/CNT/GCE	0.001–0.1 ng/mL & 0.1–30 ng/mL	0.5 pg/mL	[47]
organohydrogels	0.41–8.3 U/mL & 8.3–249 U/mL	0.805 μU/mL	[39]
Co(bpy)33+/MWNTs-Nafion/GC	1–30 U/mL & 30–150 U/mL	0.36 U/mL	[48]

**Figure 3:**
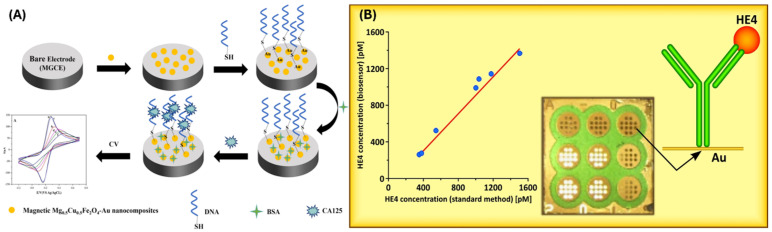
Schematics of the fabrication methods of electrochemical sensors for (**A**) CA-125 (reproduced from Yue et al., 2023, used under a Creative Commons BY-NC-SA 4.0 license) [49] and (**B**) HE4 (reproduced from Szymanska et al., 2021, used under a Creative Commons CC BY 4.0 license) [59].

For label-free detection of CA-125, Yue et al. prepared an electrochemical biosensor exploiting magnetic field-induced self-organization of nanoparticles (Figure 3A) [49]. Huang et al. utilized the surface chemistry of MXene and synthesized several MXene/Ag nanocomposites. These included Ti_3_C_2_/Ag, V_2_C/Ag, and Nb_2_C/Ag. MXene acted as the substrate for the in situ growth of Ag nanoparticles through the self-reduction of Ag(NH_3_)_2_^+^. Among these, V_2_C/Ag demonstrated the best self-reducing capability because of having several variable valence states, a bigger interlayer spacing, and more reactive groups. Additionally, it displayed notable photothermal properties and catalytic activity on oxygen reduction. Consequently, an electrochemiluminescence-photothermal (ECL-photothermal) immunosensor was created utilizing V_2_C/Ag as the ECL anchor and photothermal reagent. This immunosensor is capable of detecting lipolysis-stimulated lipoprotein receptor (LSR) (LOD was 1.34 × 10^−6^ ng/mL), which is a biomarker of ovarian cancer [50].

MiR-21 microRNA is upregulated in many cancers, including ovarian cancer. Zoughi et al. prepared a novel DNA-based biosensor for detecting miR-21 with high sensitivity and specificity. This biosensor employs silver nanoclusters (AgNCs) on a fluorescent hairpin probe. The design incorporates a poly-cytosine motif on the probe to facilitate AgNC formation and a guanine-rich sequence to increase the detection signal. The fluorescence intensity increases in direct correlation with miR-21 concentration. The developed biosensor exhibited a linear detection range for miR-21 concentrations from 9 pM to 1.55 nM. It has a detection limit of 2 pM. This sensor demonstrated high selectivity, as it did not respond significantly to other nucleic acids. It was also effective in detecting miR-21 present in human serum as well as cancer cell lines from lung and ovarian origins [51]. Zoughi’s fluorescent nucleic acid sensor introduces a shift from antibody–antigen recognition (immunosensors) to nucleic acid hybridization.

Ren et al. constructed an advanced sandwich-type immunosensor for detecting CA-125. This immunosensor’s electrochemical response correlated with CA-125 concentration. It demonstrated high sensitivity, a broad linear detection range (0.01 to 100 U/mL), and a low detection limit of 0.00417 U/mL. This design incorporated three-dimensional reduced graphene oxide that was functionalized with the amino group (3D-rGOF-NH_2_). This material exhibited high conductivity, large surface area, and stability. MgAl layered double hydroxide nanocomposites with ordered mesoporous carbon and ferrocene carboxylic acids enhanced antibody immobilization and signal amplification of this sensor [52].

Another novel electrochemical chemosensor was prepared by Can et al. to detect CA-125 rapidly and reliably. They utilized polypyrrole-based molecularly imprinted polymer surfaces. Polypyrrole is a conducting polymer. Using vapor deposition polymerization, CA-125 was imprinted on polypyrrole nanotubes (MI-PPy NT). These nanotubes were immobilized onto screen-printed gold electrodes (Au-SPE). The MI-PPy NT@Au-SPE sensor demonstrated excellent sensitivity (68.57 μA per decade) to CA-125 (0.1 U mL^−1^ to 100 U mL^−1^). The LOD was 0.4 U mL^−1^ and the correlation coefficient was 0.9922. Better sensitivity and low LOD make this sensor a promising tool for ovarian cancer diagnosis [53]. A photoelectrochemical immunoassay for CA-125 was created by Lin et al. They employed hollow porous In_2_O_3_ nanotubes containing CdS nanoparticles as the photoactive material. The surface was decorated with a capture antibody for CA-125. When CA-125 was present, a horseradish peroxidase (HRP) labeled detection antibody bound to the other side of CA-125, thus forming a sandwich immunocomplex. HRP led to the catalytic conversion of 4-chloro-1-naphthol to insoluble benzo-4-chlorohexadienone, which precipitated on In_2_O_3_/CdS. This blocked light absorption and reduced the photocurrent. The assay can detect CA-125 in the 0.1–100 U mL^−1^ range with a LOD of 0.046 U mL^−1^ (S/N ratio = 3:1) [54]. Wang and colleagues developed another photoelectrochemical immunosensor based on a CdS/Bi_2_S_3_/NiS heterostructure for CA-125 detection. The CdS/Bi_2_S_3_/NiS composite was synthesized via a hydrothermal method. The CA-125 antibody was linked using thioglycolic acid. The photocurrent response of this sensor was 14.6 times higher than that of pure CdS. It exhibited a wide linear detection range (1 pg mL^−1^–50 ng mL^−1^) and low LOD (0.85 pg mL^−1^). It also has good stability, reproducibility, and anti-interference properties [55].

**Figure 4:**
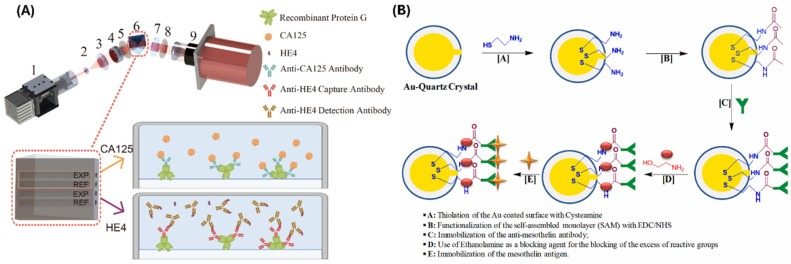
Schematics showing the fabrication methods of (**A**) an immunosensor capable of detecting both CA-125 and HE4. Here, 1 is the light source, 2 and 3 are the beam-expanding lenses, 4 is the aperture, 5 and 8 are the polarizers, 6 contains the prism and the chip, 7 is the quarter-wave plate, and 9 is the CCD camera (Reproduced from Wang et al., 2025, used under a Creative Commons CC BY 4.0 license [60]) and (**B**) a QCM-based sensor for highly sensitive detection of mesothelin (Reproduced from Joshi et al., 2023, used under a Creative Commons CC BY 4.0 license [62]).

Maghiani and colleagues functionalized NiFe_2_O_4_ with cystamine, glutaraldehyde, and polyvinylidene fluoride and developed an immunosensor against CA-125 for point of care [56]. Yılmaz and Bilgi have prepared a label-free impedimetric CA-125 immunosensor using screen-printed carbon electrodes. They have modified these electrodes with poly(toluidine blue) (PTB) and gold nanoparticles (AuNPs). This immunosensor showed a LOD of 1.20 pg mL^−1^ and a linear range of 5–100 pg mL^−1^. The synergistic interaction between PTB and AuNPs enhanced the electrochemical and analytical performance of this immunosensor [57]. Human epididymis protein 4 (HE4) is an early-stage biomarker of ovarian cancer. Xu and associates engineered a sandwich immunosensor for detecting HE4. Titanium metal–organic framework (TiMOF) modified with ketjen black (KB) and AuNPs enhances electron transfer and antibody immobilization. TiMOF was fixed on an electrode made of glassy carbon. HE4 binds with the antibody on TiMOF. Prussian blue (PB) bound to labeled antibodies provides strong electrochemical signals upon binding with HE4. This approach achieves high sensitivity, with a fairly wide linear range (0.1–80 ng mL^−1^) and a low LOD of 0.02 ng mL^−1^ [58]. Szymanska et al. fabricated a biosensor utilizing the surface plasmon resonance imaging technique for detecting HE4 in plasma label-free (Figure 3B) [59], and Wang et al. fabricated an immunosensor capable of detecting both CA-125 and HE4 (Figure 4A) [60].

Lysophosphatidic acid (LPA) is another early-stage ovarian cancer biomarker. When LPA binds to the gelsolin–actin protein complex, decoupling of this complex occurs. Davoudian and colleagues prepared 2 types of sensors based on this complex for detecting LPA- one is an electromagnetic, piezoelectric, and acoustic sensor, and the other is a chemiluminescence sensor based on Fe_3_O_4_ nanoparticles [61].

Collectively, biosensors for ovarian cancer detection have evolved toward higher sensitivity, selectivity, and miniaturization. Electrochemical platforms provide excellent sensitivity and reproducibility, while photoelectrochemical and luminescent systems offer dual-modality detection and visual outputs. The expansion beyond CA-125 to alternative biomarkers (HE4 and LPA) addresses a fundamental limitation of single-marker (CA-125) diagnostics, since CA-125 elevation occurs in only 50–60% of early-stage ovarian cancers. Future clinical diagnostics will likely employ sensor arrays detecting multiple ovarian cancer markers simultaneously to improve diagnostic accuracy. Reproducibility, simpler fabrication, and lower cost are some critical factors that influence the trade-off between sensitivity and practical implementation.

### 2.3. Microfluidic Devices & Ovarian Tissue Engineering

**Figure 5:**
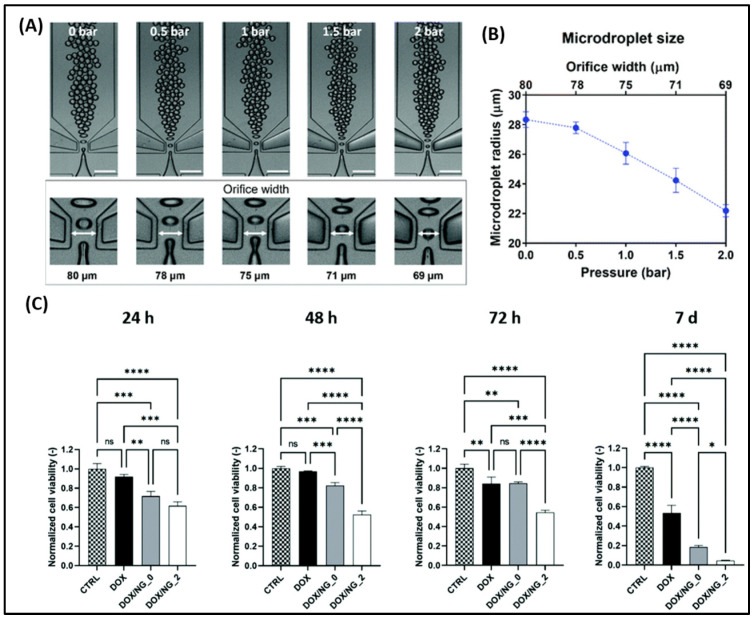
(**A**) Microfluidic nanogel production at different orifice widths and actuation pressures. (**B**) The largest nanogel droplet was formed at 0 bar (NG_0), and the smallest ones were formed at 2 bar (NG_2). (**C**) Cytotoxic effect on OVCA433 cells exerted by the nanogels over a period of 7 days. Cell viability levels (from MTT assay) standardized against internal controls are used to express the treatment effect. The mean ± SD of three separate experiments is the result. One-way ANOVA was used for statistical analysis (ns = not significant, * *p* < 0.05, ** *p* < 0.01, *** *p* < 0.001, and **** *p* < 0.0001). Reproduced from Giannitelli et al., 2022, used under a Creative Commons CC BY-NC 3.0 license [64].

Ovarian cancer cells often overexpress carnitine palmitoyltransferase 1A (CPT1A), a mitochondrial enzyme. Amiodarone, a known CPT1A inhibitor, was encapsulated into nanoparticles by Saorin et al. using microfluidic channels and tested on three ovarian cancer cell lines—Kuramochi, OVCAR-5, and A2780. Lipidomic changes were observed in the cells treated with amiodarone [63]. Giannitelli et al. prepared microfluidic-based droplet nanogels from hyaluronic acid and polyethyleneimine to deliver doxorubicin (Figure 5A,B). These nanogels showed significant toxicity against OVCA433 cells when compared with free doxorubicin (Figure 5C). The polydispersity index of the nanogels prepared in this method was very low (0.015). Such low variation would not otherwise be achievable using conventional methods [64]. De La Rosa et al. prepared PEGylated PLGA nanoparticles and surface modified the nanoparticles with 2 different types of RGD peptide—cyclic RGD (has a preferential binding affinity to α_v_β_3_ integrin) and linear RGD (has similar affinity to both α_v_β_3_ and α_5_β_1_ integrins). A microfluidic-assisted nanoprecipitation technique was adopted. They showed nanoparticles with cyclic RGD peptide accumulated more inside the U87MG cells (glioma cell line that highly expresses α_v_β_3_ and moderately/highly expresses α_5_β_1_) in comparison with the A2780 ovarian cancer cell line (almost nil α_v_β_3_, moderately expresses α_5_β_1_). However, the linear RGD was uptaken equally by both cell lines. This suggests the non-selective affinity of linear RGD to integrins. No superiority of cyclic or linear RGD in nanoparticle accumulation in A2780 cells was observed [65].

These studies demonstrate that microfluidic fabrication exerts superior control over nanoparticle properties. For instance, Giannitelli’s platform achieved remarkably low polydispersity (0.015). With careful targeting strategies, microfluidic-based nanoparticles can offer uniform formulations.

**Figure 6:**
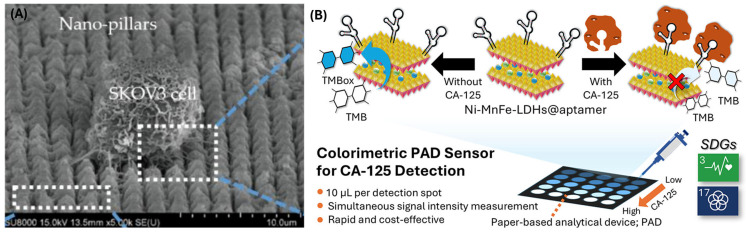
(**A**) SKOV3 cell capture by the microfluidic platform developed by Jou et al., 2021, reproduced under a Creative Commons BY-NC-SA 4.0 license [67]. (**B**) Paper-based platform fabricated for detecting CA-125 antigen by Prakobkij et al., 2025, reproduced under a Creative Commons CC BY-NC-ND 4.0 license [71].

Micro RNA-21 (miRNA-21) is expressed in many cancers, including ovarian cancer. Sung et al. developed a microfluidic platform for detecting miRNA-21 by digital polymerase chain reaction. The limit of detection of this platform is 11 copies of miRNA per mL. The inaccuracy rate of detection is <12% [66]. Utilizing anti-EpCAM (anti-epithelial cell adhesion molecule) and anti-N-cadherin antibodies, Jou and coworkers developed an automatic microfluidic platform for detecting circulating ovarian cancer cells (Figure 6A). The sensitivity and specificity of this platform were 62.5% and 100%, respectively. The limitation of this device is that the sample size of patients and healthy controls tested was small [67]. Using a commercial microfluidic system for capturing and analyzing cells (Cell Reveal^TM^, Taipei City, Taiwan), Chang et al. compared the genomics of epithelial ovarian cancer and circulating tumor cells (CTCs). They found that genomic mutations in the CTCs are not always the same in the primary tumor cells. This finding deserves further research to reveal the mechanism of CTC formation and the factors behind their genomic differences with the primary cancer cells [68].

Saadati et al. invented a microfluidic platform for detecting CA-125 in plasma. They deposited silver—D-penicillamine—graphene quantum dots (Ag–DPA–GQD) nano ink on an electrode made of glassy carbon. Cysteamine (CysA)-gold nanoparticles were deposited on this nano-ink using the chronoamperometry technique. Activated anti-CA-125 monoclonal antibodies were fixed on the nano-ink surface as the capturing antibody. The linear range obtained for this biosensor was 0.001–400 U mL^−1^, and the low limit of quantification (LLOQ) was 0.001 U mL^−1^ [69]. A similar paper-based microfluidic biosensor was created by Bahavarnia et al. They deposited silver/reduced graphene oxide nano-ink on a photographic paper by handwriting. Then, CysA/Au nanoparticles were covalently conjugated to the ink, and the anti-CA-125 antibody was immobilized. The linear range of this biosensor was 0.78–400 U/mL, whereas the LLOQ was 0.78 U/mL [70]. Prakobkij et al. conjugated an aptamer to Ni-MnFe-layered double hydroxides and developed a paper-based platform for colorimetric detection of CA-125 (Figure 6B) [71].

Wimalachandra et al. developed CCL21-folic acid-loaded upconversion nanoparticles. They wanted to study how these nanoparticles are uptaken by and later attract immune cells to the tumor site. They built a 3D microfluidic platform to mimic the endothelial-tumor structure and used this platform to study the extravasation of dendritic and Jurkat T cells across the endothelial barrier to the OVCAR-3 cells [72]. Zhang et al. fabricated a three-dimensional, nanopatterned microfluidic chip to produce colloids that would self-assemble to build a platform for detecting tumor-related exosomes. This platform is capable of detecting 10 exosomes μL^−1^ of plasma, a performance superior to commercially available alternatives at the time their research was published. Their findings suggest folate receptors present in these exosomes can be utilized as an early biomarker of ovarian cancer [73]. After capturing exosomes, releasing them without physical alteration and label-free is challenging. Hisey et al. fabricated a herringbone-patterned microfluidic device and functionalized it with CD9 and EpCAM to capture exosomes present in the serum of high-grade serous ovarian cancer patients. The captured exosomes could then be released unchanged using a buffer of low pH and collected for further characterization [74].

Microfluidic devices enable real-time, multiplexed, and miniaturized analyses. Compared to paper-based systems, microfluidic channels provide superior precision, dynamic flow control, and integration with nanomaterials. Thus, they allow for the detection of a wide range of entities—cells, biomarkers, miRNA, and exosomes. Paper-based systems, however, offer affordability, disposability, and ease of use for field diagnostics.

Another approach is quartz crystal microbalance (QCM)-based immunosensors for highly sensitive, label-free detection of ovarian cancer biomarkers. These sensors can measure mass changes at a scale of picograms to nanograms on a piezoelectric crystal surface. Joshi et al. developed a QCM-based immunosensor for detecting mesothelin at a linear range of 100 pg/mL-50 ng/mL (Figure 4B) [62] while Chen et al. fabricated a QCM immunosensor for robust detection of HE4, CEA, CA125, CA19-9, and AFP [75].

Biomaterials can also help preserve follicles. Tissue-engineered ovaries can help restore fertility in cancer patients where there are risks of damage to ovarian follicles by radiotherapy and chemotherapy. In an attempt to build a tissue-engineered ovary, Dadashzadeh and colleagues prepared a hydrogel using PEGylated fibrin. They isolated stromal cells from the ovary and successfully cultured them on this hydrogel. Since stromal cells are supporting cells of the ovary, based on their behavior, the authors hypothesized that this hydrogel is a potential solution for encapsulating and preserving preantral ovarian follicles [76]. In a similar effort, Kim and colleagues prepared a hydrogel matrix from PEGylated vinyl sulfone. They isolated follicles enzymatically from the ovaries of mice aged 6–7 days and encapsulated them in the hydrogel. They then placed the hydrogel orthotopically in ovariectomized mice. At 30 days, multiple matured antral follicles, as well as corpora lutea, were observed. These findings correspond with the normal ovulation cycle. Vascularization was also observed in the implanted graft. Based on the morphology of follicles, graft neovascularization, and the hormonal level of FSH, the authors concluded that the developed hydrogel is capable of functioning as an artificial ovary for at least 60 days [77]. Bioinks with suitable viscosity, adjustable biophysical and biochemical properties, and good biocompatibility are crucial for creating tumor models that mimic native tumors. Wang and colleagues developed a nanocomposite hydrogel bioink from aldehyde-modified hyaluronic acid (aHA), aldehyde-modified cellulose nanocrystals (aCNCs), and gelatin for 3D bioprinting of ovarian and colon cancer models. Properties such as mechanical properties, gelation time, and printability of this hydrogel can be regulated by varying the ratio of aCNCs to gelatin [78]. These studies demonstrate tissue engineering approaches can help preserve follicles and build 3d disease models to gain a better insight.

## 3. Applications of Biomaterials in Ovarian Cancer Treatment

### 3.1. Nanoscale Drug Delivery Systems (DDSs) for Ovarian Cancer

#### 3.1.1. Targeted and Stimuli-Responsive Drug Delivery Systems

Different ovarian carcinoma histotypes show distinct, overexpressed cell-surface signatures that make these attractive targets for antibody- or ligand-directed therapies. HGSOC frequently displays folate receptor-α, MUC16 (CA-125), EpCAM, and often mesothelin. Low-grade serous carcinoma can also express FRα and EpCAM, but at lower/variable rates than HGSOC [79,80,81,82]. Clear-cell carcinoma commonly shows mesothelin and integrin (αvβ3/αvβ5) pathway activity and may harbor HER2 overexpression in a minority [79,83]. Endometrioid tumors often express MUC16/CA-125 and occasionally show HER2 positivity, while mucinous carcinomas have the highest incidence of ERBB2/HER2 amplification among ovarian cancer histotypes [81,84]. Finally, molecules involved in peritoneal dissemination, such as the urokinase receptor (uPAR) and chemokine receptor CXCR4, are enriched in tumors with peritoneal/ascitic spread, and they are under investigation as targets to limit metastasis and improve intraperitoneal delivery [85,86,87].

The majority of ovarian cancer tissues have high levels of somatostatin receptor 2 (SSTR2) on the cell membrane. A widely used somatostatin analog, octreotide, enters cancer tissue through SSTR2. Liu et al. modified liposomes with octreotide to actively target SKOV3 cells via SSTR-mediated endocytosis and extend the drugs’ (paclitaxel and neferine) circulation time in vivo [88].

Tang and colleagues encapsulated doxorubicin (DOX) within dual-functionalized mesoporous silica nanobeans (DF-MSNB). This complex releases the drug and tracks its uptake by cells through an environmentally triggered mechanism. In the microenvironment of tumor tissues, elevated glutathione (GSH) levels and reduced pH break the disulfide bond of the linker, thus activating a fluorescent group and releasing DOX. The results showed DOX@DF-MSNB localized within lysosomes and mitochondria in ovarian cancer cells (A2780), causing mitochondrial dysfunction and activating mitochondria and macrophage-mediated autophagy [89]. Colli and colleagues synthesized nanoparticles from polymers with an upper critical solution temperature in order to deliver paclitaxel to SKOV3 ovarian carcinoma cells and release it upon reaching a hyperthermic state. These nanoparticles were created using the RAFT polymerization process. This process involved the self-assembly of poly(sulfobetaine-co-sulfabetaine) (p(SB-co-ZB)) copolymers with a hydrophobic block of vinyl oligoesters. The nanoparticles released nearly all the entrapped drug after an hour at 43 °C while retaining over 95% of the medication at the physiological temperature (37 °C) [90].

Supramolecular organic frameworks (SOF) come into existence when organic molecules self-assemble via non-covalent interactions, such as hydrogen bonding, van der Waals forces, π-π stacking, and host-guest interactions. Zhang et al. developed an SOF by self-assembling a photosensitive molecule with cucurbit [8] uril. This photosensitive molecule was OTPP-6-Methyl. This approach enhances its dim fluorescence, which is caused by restricted molecular motion. While OTPP-6-Methyl binds to different negatively charged molecules because of its high positive charge, this SOF only shows a significant fluorescence signal when lysophosphatidic acid (LPA) is present. LPA is an ovarian malignancy biomarker, and the signal is emitted because of SOF disassembly and subsequent binding with LPA. To further enhance the fluorescence changes, a fluorescence resonance energy transfer (FRET) system with Cyanine 5 was introduced. Additionally, a tumor-targeting cyclic RGD peptide group was included in a guest molecule (OTPP-5-M−1−cRGD) to enhance specificity. The SOF improves the photosensitivity of guest molecules, enabling imaging of the subcutaneous models of ovarian tumor and serving as a DDS for combined chemotherapy and photodynamic therapy with doxorubicin [91].

**Figure 7:**
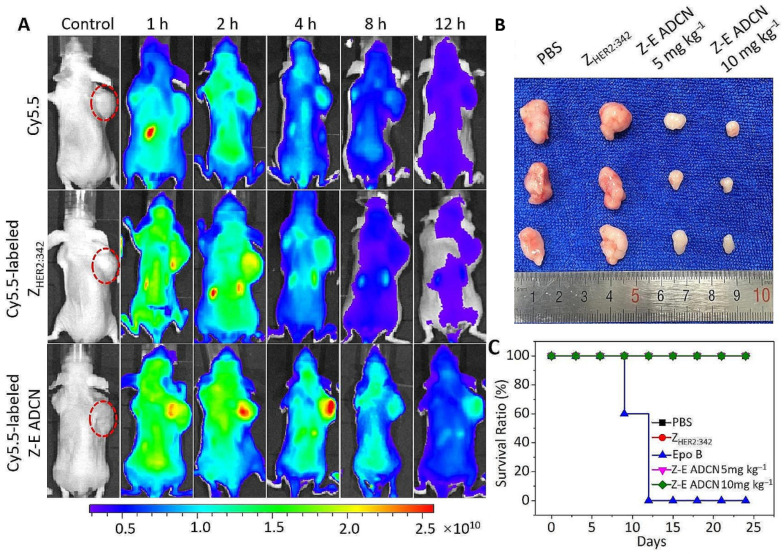
(**A**) Biodistribution of free Cy5.5 dye, Cy5.5-labeled ZHER2:342, and Cy5.5-labeled Z-E ADCN, (**B**) tumor volumes after 24 days of treatment, and (**C**) survival curves of different groups of tumor-bearing mice. Reproduced from Xia et al., 2024, used under a Creative Commons CC BY-NC-ND 4.0 license [92].

Epothilone B (Epo B) is a potent antineoplastic compound, but its clinical application is hindered by poor tumor selectivity and limited therapeutic windows. Non-immunoglobulin affinity proteins called affibodies provide accurate targeting to cancer cells’ overexpressed receptors, making them ideal for enhancing drug delivery. Xia et al. developed a novel nanoagent, ZHER2:342-Epo B Affibody-Drug Conjugate Nanoagent (Z-E ADCN). It linked an affibody targeting the HER2 receptor with Epo B with the help of a reactive ROS-sensitive thioketal group. This design ensured Epo B was encapsulated within an affibody corona, reducing side effects on normal tissues. Z-E ADCN demonstrated precision targeting ability and sufficient accumulation in tumors, as the HER2-specific affibody facilitated endocytosis. Upon internalization, Epo B was released because of elevated ROS levels within cancer cells, exhibiting strong anticancer effects in HER2-positive tumor models. Because of the properties at the nanoscale, the circulation time and tumor retention of these nanoagents were extended. This minimized adverse effects on other normal organs (Figure 7) [92].

Ginsenoside RG3 is an active component of ginseng that has demonstrated significant antitumor properties. However, oral administration of RG3 results in low drug concentrations at the tumor site, particularly in ovarian cancer (OC). Yi and colleagues explored the use of microneedles (MNs) made from methacryloyl gelatin to directly deliver RG3 to ovarian tumors. The authors found RG3-loaded MNs successfully penetrated tumor cells and released the cargo with optimal kinetics, resulting in inhibition and apoptosis of OC cells. Both in vitro and in vivo experiments supported the efficacy as well as biosafety of RG3-MNs. This drug delivery method bypasses the first-pass hepatic metabolism and degradation in the gastrointestinal tract, which are associated with oral administration, enhancing drug delivery efficiency [93].

It is possible to accumulate drug-loaded nanoparticles at the target site by applying a magnetic field. Magnetic mesoporous silica nanoparticles were loaded with cis-diaminodichloroplatinum (Fe_3_O_4_@SiO2-CDDP) for enhanced ovarian tumor penetration by Chen et al. A hybrid torque-force magnetic field actively transported these nanoparticles to the tumor location. These nanoparticles showed deep penetration and released the drug in response to the tumor’s acidic environment. Cytotoxicity of these nanoparticles was assessed in both 3D spheroids and mouse models. These nanoparticles showed enhanced drug concentration locally and reduced adverse effects compared to traditional chemotherapy [94]. pH-responsive polymeric micelles are valuable for delivering cytotoxic drugs to specific target sites, especially in cancer tissues where the pH is usually low. Oligoelectrolyte-mediated, pH-responsive micelles against ovarian cancer cells were prepared by Hussain and colleagues. They used oligo(2-vinyl pyridine) (OVP) with PEG-b-PCL micelles. Dynamic light scattering (DLS) showed that OVP increased micelle size at pH 7.4 but decreased it below pH 6.5 due to OVP release from the micelles. Satisfactory results were achieved by co-encapsulating drugs (doxorubicin, paclitaxel, gossypol, or 7-ethyl-10-hydroxycamptothecin (SN38)) with OVP in the micelles. OVP-loaded PEG10PCL10 (long hydrophobic block) micelles exhibited rapid drug release triggered by pH, according to in vitro release tests. This release was hastened by increased OVP loadings. No triggered release was seen in PEG4PCL4 (short hydrophobic block) micelles, indicating the importance of molecular weight. Metabolic assays indicated that drug- and OVP-loaded PEG10PCL10 micelles had higher cytotoxicity than those drug-loaded micelles without OVP. Lower OVP loadings were nearly as effective as higher loadings in terms of cellular metabolic activities, although with significant differences in the drug-releasing behaviors. These findings highlight the potential of oligoelectrolyte-mediated, pH-triggered drug release in enhancing the efficacy of pH-responsive micelles. The limitation of this study is that no in vivo experiments were conducted [95].

A liposomal delivery system was developed by Juul et al. for efficient oxaliplatin delivery to HER2-positive tumors. Trastuzumab on the liposome surface targets HER2 receptors and enhances the internalization of liposomes. To evade uptake by the mononuclear phagocyte system, an outer layer of PEG was added. A peptide linker sensitive to protease was added to the base of every PEG molecule. Upon cleavage of this linker by matrix metalloproteinases (MMPs) in tumors and subsequent removal of the PEG layer, the liposomes become destabilized and release oxaliplatin [96]. Extracellular vesicles (EV) are a promising natural drug carrier. However, they lack specificity, much needed for targeted drug delivery. Ephrin-B4 is a receptor that is overexpressed in ovarian cancer. Ephrin-B2 is a ligand of the ephrin-B4 receptor. The expression of ephrin-B2 with another EV membrane protein, LAMP2b, resulted in more efficient internalization of EV in ovarian cancer cells than unaltered EV. This trend was observed both in vitro and in vivo [97]. Incorporating tetramethylpyrazine (TMP) into exosomes demonstrated anti-ovarian cancer activity against A2780T cells and enhanced paclitaxel (PTX) efficacy. Reversal of drug resistance was achieved by downregulating multidrug resistance protein 1, multidrug resistance-associated protein 1, and glutathione S-transferase Pi expression. These PTX-loaded exosomes significantly increased apoptosis in the cancer cells that previously achieved drug resistance [98].

The aforementioned studies reveal three fundamental principles for effective ovarian cancer drug delivery: the critical importance of exploiting tumor microenvironment characteristics (pH, GSH, ROS, temperature), the superiority of multi-trigger responsive systems over single-stimulus designs, and the necessity of matching the delivery platform to the drug’s physicochemical properties. When compared, receptor-mediated targeting (SSTR2, HER2, ephrin-B4, CD44) consistently outperforms passive accumulation strategies across different nanocarrier types. However, the optimal targeting ligand varies with tumor heterogeneity. Perhaps most significantly, systems combining diagnostic capabilities with therapeutic functions (theranostic platforms) demonstrate that future developments would integrate imaging to enable real-time monitoring of drug delivery efficiency. Successful translation of any novel system would require balancing targeting specificity, stimulus-responsiveness, cargo compatibility, biocompatibility, and manufacturing scalability rather than optimizing any single parameter in isolation.

#### 3.1.2. Combination Therapy and Overcoming Chemoresistance

Sphingosine Kinase 1 (SphK1) regulates pro-survival pathways in ovarian cancer. Wang et al. co-encapsulated PF543, a SphK1 inhibitor, and carboplatin (CBP) within PLGA nanoparticles to simultaneously deliver both agents. In vitro as well as in vivo experiments confirmed this combinatorial delivery sensitized platinum-insensitive SKOV3 cells to CBP, suggesting a viable strategy to overcome chemoresistance [99]. Gaikwad et al. developed a nanomicelle system containing 2 drugs- albendazole and paclitaxel. The nanomicelles were prepared from D-tocopheryl polyethylene glycol 1000 succinate (TPGS) and Soluplus^®^ and modified with folic acid using the solvent evaporation technique. The optimal formulation showed a critical micelle concentration (CMC) of 0.0015 mg/mL, which indicated good dilution stability. Various characterization techniques, such as DSC, NMR, and FTIR, confirmed the micelles’ stability and drug compatibility. These micelles’ release profiles included a burst at the beginning and a continuous release over the course of ninety hours. Superior cytotoxicity was observed for these micelles compared to the free drugs alone. In vivo studies showed effective targeting and penetration, with both drugs present in plasma and tumor tissues. However, a limitation of this study is that antitumor activity was assessed only for 15 days in a mouse model of ovarian tumor [100].

BACH1, a protein highly expressed in ovarian tumors, promotes migration, metastasis, poor prognosis, and drug resistance in ovarian cancer. Similarly, CD47 is upregulated and associated with poor prognosis by inhibiting macrophage phagocytosis of ovarian tumor cells. BACH1 and CD47 levels are positively correlated. Heme, an inhibitor of BACH1, can restore chemotherapy sensitivity but is unstable. To address this, nanoparticles combining heme and cisplatin (DDP) with folic acid for targeting were developed by Gong and colleagues. These nanoparticles inhibit BACH1 and CD47, promote tumor cell apoptosis, enhance macrophage phagocytosis, target mitochondria, and increase reactive oxygen species (ROS) generation, thus suppressing tumors. In vivo experiments showed increased M1 macrophages in ovarian tumors. This nanoparticle-based approach offers a potential therapeutic strategy for platinum-resistant ovarian cancer by combining BACH1 inhibition and enhanced drug delivery [101].

Chemotherapy often leads to multidrug resistance (MDR) because of drug efflux caused by P-glycoprotein (P-gp). This process requires ATP hydrolysis. Inhibiting ATP production in cancer cells can potentially reverse MDR. Lu and colleagues developed PLGA-PEG nanoparticles loaded with atovaquone (ATO), which inhibits oxidative phosphorylation, quercetin (QUE), which inhibits glycolysis, and paclitaxel (PAX). PTX-ATO-QUE nanoparticles (PAQNPs) inhibit energy metabolism pathways to counteract MDR. In the in vitro experiment, it was found that PAQNPs target paclitaxel-resistant A2780/Taxol ovarian cancer cells by suppressing hexokinase II (HK II) and mitochondrial complex III activities. This resulted in a drop in the level of intracellular ATP, thus inhibiting cellular multiplication and lowering P-gp activity. Increased PTX accumulation in cells raised ROS and led to apoptosis of the resistant cells. In a mouse model with A2780/Taxol tumors, PAQNPs halted tumor growth. Immunohistochemical analysis revealed suppressed P-gp expression, thus indicating effective MDR reversal. Safety studies confirmed the suitability of PAQNPs, including stability of the serum biochemical indices, no significant toxicity, and normal organ weights. PAQNPs offer hope in overcoming MDR in ovarian cancer [102].

Tang and colleagues prepared liposomes modified with PEG and hyaluronic acid. These liposomes (PEG-TK-HA-PDLPs) were used to simultaneously deliver diosgenin and paclitaxel to ovarian cancer cells. Hyaluronic acid was utilized to target CD44. This formulation helped the liposomes have a long circulation time and aggregate at the target site in sufficient concentration [103]. To address chemoresistance, nucleic acid aptamer-modified zinc ferrite nanoparticles (NAZs) have been developed by Zhu et al. The goal was to induce autophagy and enhance cisplatin (DDP) sensitivity in ovarian tumor cells. Zinc ferrite nanoparticles triggered autophagy in SKOV3 cells. The cell death was attributed to the release of zinc ions and increased ROS production. Tumor-targeting aptamer (NucA) modification improved specificity for ovarian cancer cells. NAZs sensitized cells to DDP, showing enhanced efficacy in vitro and in vivo. Additionally, NAZs acted as T2-weighted MRI contrast agents. This dual therapeutic and diagnostic approach offers a promising way to overcome chemoresistance and attain better outcomes in ovarian cancer therapy [104].

**Figure 8:**
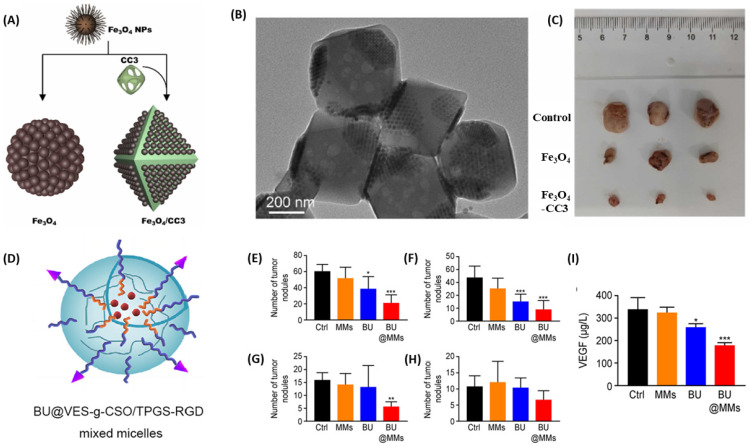
(**A**) Schematic of nanocomposites made of Fe_3_O_4_ nanoparticles and porous organic cage CC3. (**B**) Image of the nanocomposites obtained by transmission electron microscopy. (**C**) Tumor volumes in controls and after 12 days of intratumoral injection of Fe_3_O_4_ and Fe_3_O_4_-CC3 nanocomposites. Reproduced from Wei et al., 2024, used under a Creative Commons CC BY-NC-ND 4.0 license [106]. (**D**) Schematic of bufalin-loaded micelles prepared by Xu et al., 2023 [107]. The number of metastatic nodules on the 40th day in the (**E**) intraperitoneal cavity, (**F**) abdominal wall, (**G**) intestines, and (**H**) diaphragm. Concentrations of VEGF (**I**), MMP-2, and MMP-9 (not shown in the figure) were measured in different groups as markers of metastasis. The mean ± SD is the result for any group. One-way ANOVA was used for statistical analysis (ns = not significant, * *p* < 0.05, ** *p* < 0.01, and *** *p* < 0.001). Reproduced from Xu et al., 2023, used under a Creative Commons CC BY 4.0 license [107].

Bian and colleagues synthesized ferroplatinum nanoliposomes with folic acid as the targeting molecule and encapsulated DDP for the same dual purposes [105]. Wei et al. showed treatment with nanocomposites of Fe_3_O_4_ nanoparticles and porous organic cage CC3 significantly inhibited the subcutaneous transplantation model of Ovarian Cancer (SKOV3 cells) in Nude Mice by CytC/caspase-3 pathway (Figure 8A–C) [106]. Ovarian cancer very commonly metastasizes to the peritoneum. Bufalin, a natural compound, shows antitumor effects, but it is poorly soluble and toxic. Xu and coworkers prepared micelles from vitamin E succinate-grafted chitosan oligosaccharide and RGD peptide-conjugated d-alpha-tocopheryl polyethylene glycol 1000 succinate. They loaded bufalin on the micelles. In SKOV3 and A2780 cells, these micelles decreased migration and invasion, caused apoptosis, and hindered cell growth. They have been reported to reduce tumor burden in an intraperitoneal metastasis model (Figure 8D–I) [107].

A comparative analysis of these combination strategies reveals that successful chemoresistance reversal requires targeting the specific molecular mechanisms underlying resistance rather than simply increasing drug dosage. The metabolic inhibition approach (PAQNPs targeting both glycolysis and oxidative phosphorylation pathways) proved more robust than single-pathway interventions. Also, platforms that simultaneously modulate the tumor immune microenvironment (such as heme-cisplatin nanoparticles increasing M1 macrophages) demonstrated superior efficacy compared to those targeting cancer cells alone. This suggests that future combination therapies must account for the complex relationship between malignant cells and their environment. Overcoming chemoresistance needs rational drug combinations based on a comprehensive understanding of the underlying resistance pathways. Nanocarriers can be the enablers of simultaneous multi-target engagement in this context. A summary of the discussions in Section 3.1.1 and Section 3.1.2 can be found in Table 2.

#### 3.1.3. Immunomodulatory and Gene-Based Therapies

Cell membrane-camouflaged nanoparticles were created by Chen et al. for synergistic delivery of chemotherapy and genes in ovarian cancer. These nanoparticles contained mitoxantrone (Mito) and Her2 antisense oligonucleotides (Her2 ASOs). A hybrid membrane made of SKOV3 cells and red blood cells covered the nanoparticles. The RBC membrane helped to evade the immune system and achieve a long circulation time. The cancer cell membrane enabled tumor targeting. The system achieved 75.7% apoptosis in vitro and 83.3% tumor suppression in vivo [108]. Han et al. developed a tubular DNA origami that is compact, controllable, and can effectively protect cargo. They used fluorescent-labeled DNA strands as cargoes that can bind to microRNA 21 (miR-21) overexpressed in SKOV3/DDP and A2780/DDP ovarian cancer cells. The DTO was successfully internalized in ovarian tumor cells and caused a significant decrease in miR-21 expression. DTO is a promising nanocarrier for cancer treatment and biosensor development [109].

The human epidermal growth factor receptor 2 (HER2), a transmembrane tyrosine kinase receptor, is overexpressed in various cancers, including gastric, ovarian, and breast cancers. HER2 overexpression correlates with more tumor aggressiveness, unfavorable prognosis, and shorter survival time. Although several HER2-targeted medications exist, relapses are common. For HER2-positive tumors, Hu and associates created a lipid nanoparticle (LNP)-based treatment that encapsulated an mRNA encoding a new HER2-CD3-Fc bispecific antibody (bsAb). The bsAb, which interacts with both HER2 and CD3 with high affinity, was robustly secreted by the LNPs after they successfully transfected ovarian cancer cells such as SKOV-3 and A1847. Interaction with CD3 was essential for T-cell involvement and antitumor activity since its ablation led to the loss of function of bsAb. bsAb caused strong T cell-directed cytotoxicity against HER2-positive tumor cells. The anticancer effect observed was exclusively due to HER2 binding, as HER2 knockout cells gained resistance, and HER2-overexpressing cells demonstrated sensitivity to the bsAb. In vivo, intratumoral injection of LNPs containing HER2-CD3-Fc mRNA stopped the growth of HER2-positive cells in a rodent ovarian cancer model without toxicity. These findings suggest that this LNP-based treatment may be able to safely and successfully treat HER2-positive cancers [110].

Chemodynamic therapy (CDT) is a relatively new approach where the Fenton or a Fenton-like reaction is utilized to obtain •OH in the cancerous tissue that results in cellular death as well as the release of antigens associated with tumors. However, it alone is often insufficient for a robust immune response. To improve CDT and increase antitumor immunity, Xi et al. presented FeS nanoparticles made on the surface of *Shewanella oneidensis* MR-1 bacteria (FeS@MR-1). In acidic environments, FeS@MR-1 degrades, releasing H_2_S and Fe(II) ions. H_2_S inhibits catalase enzyme, leading to H_2_O_2_ accumulation and toxic ·OH radical generation via Fenton reaction, whereas Fe(II) acts as the Fenton catalyst. Pathogen-associated molecular patterns of *S. oneidensis* function as immune adjuvants by activating dendritic cells (DCs) and other immune cells, thus reversing the immunosuppressive microenvironment of the malignant tissue. FeS@MR-1 triggers systemic antitumor responses against primary, distant, and orthotopic ovarian tumors [111]. One major limitation of CDT is the glutathione (GSH)-mediated scavenging of free radicals. To address this, extracellular vesicle mimetic nanovesicles loaded with β-Lapachone (Lapa) and iron oxide nanoparticles (IONPs) were prepared by Wang et al. Catalysis of Lapa by NADPH quinone oxidoreductase 1 generated H_2_O_2_, while iron from IONPs in the acidic environment converted H_2_O_2_ into toxic •OH via Fenton reaction. Additionally, Lapa depleted tumor GSH, further intensifying oxidative stress [112].

An injectable nanohydrogel combining carboxymethyl chitosan (CMCS) and polylactic acid-hyperbranched polyglycerol bioadhesive nanoparticles (BNPs) was developed by Liang et al. for treating intraperitoneal ovarian cancer metastasis. Paclitaxel was loaded on BNP, and anti-PD-1 antibodies were loaded on the CMCS network. BNPs and CMCS were crosslinked via reversible Schiff base bonds. This system showed sustained release of the drug over seven days. In a mouse model, this drug delivery system effectively suppressed ovarian cancer peritoneal metastasis, demonstrating superior therapeutic potential compared to other groups [113]. Wu and associates developed a DDS targeting the human epidermal growth factor receptor (EGFR). It is overexpressed in ovarian cancer cells. They first loaded doxorubicin into mesoporous silica nanoparticles. Then, they modified these nanoparticles with human serum albumin (HSA) and finally conjugated humanized single-chain antibody fragment (husA) to HSA. husA minimized immunogenicity while enhancing tumor penetration because of its small size and lack of Fc-induced effects. HSA improved nanoparticle biocompatibility and maintained prolonged circulation. It also enabled enzyme/pH-responsive doxorubicin release via matrix metalloproteinase 2 (MMP-2)/low pH degradation. A high drug-to-antibody ratio of 11.8 for this formulation surpasses other currently available antibody-drug conjugate drugs [114].

**Figure 9:**
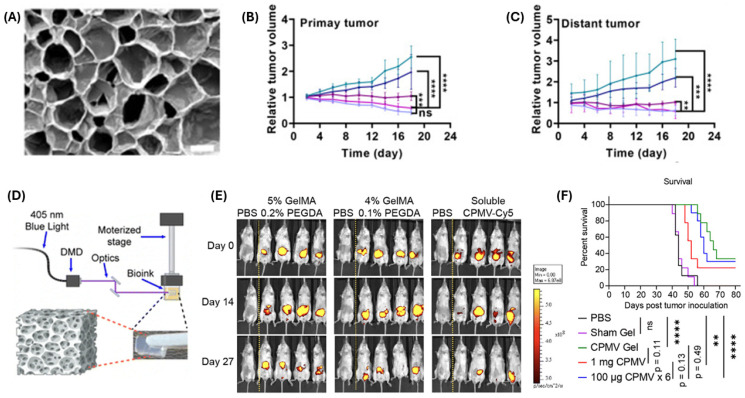
(**A**) SEM image of the nanogel prepared by Li et al., 2024 [115]. The white scale bar corresponds to 50 μm. (**B**) Relative volumes of tumors at the primary sites and (**C**) distant sites (representative of metastasis). The mean ± SD of five separate experiments is the result. One-way ANOVA or *t*-test was used for statistical analysis (ns = not significant, ** *p* < 0.01, *** *p* < 0.001, and **** *p* < 0.0001. Reproduced from Li et al., 2024, used under a Creative Commons CC BY-NC-ND 4.0 license [115]. (**D**) Schematic showing the CPMV implant bioprinting process, (**E**) fluorescent CPMV-Cy5 implants imaged on days 0, 14, and 27, (**F**) survival rates of different treatment groups. Reproduced from Zhao et al., 2024, used under a Creative Commons CC BY-NC 3.0 license [116].

Tumor vaccine, a recent addition to the treatment arsenal against cancer, induces immune responses using antigen(s) from the tumor itself. A smart nanogel system was developed by Li et al. to create an in situ nano-vaccine for ovarian cancer treatment. They first prepared PLGA nanoparticles that were later modified with mannose. They then encapsulated CpG, a synthetic DNA molecule that acts as an immune adjuvant (CpG@Man-P). Mannose was incorporated for binding with dendritic cells and CpG for activating TLR9. An MMP-2-responsive gel co-encapsulated CpG@Man-P and trametinib (Tra) to enable continuous generation of antigens, lymph node drainage, and immune activation. The 40 nm CpG@Man-P demonstrated superiority in terms of cargo release, lymph node accumulation, and antitumor effects. With the addition of PD-1 antibody, this vaccine significantly suppressed growth and metastasis in an ovarian cancer model (Figure 9A–C) [115].

Cowpea mosaic virus (CPMV) induces significant antitumor immune responses in ovarian cancer. It acts as a toll-like receptor (TLR) agonist (TLR 2, 4, 7) and activates innate immune cells at tumor sites. These cells mediate tumor killing and initiate adaptive immunity. Repeated intraperitoneal injections are typically required to maintain the immunogenicity of CPMV. To address this, implantable CPMV-loaded slow-release hydrogel depots were developed by Zhao et al. Using digital light processing (DLP) printing, hydrogels were fabricated with GelMA and PEGDA bioink containing 900 μg CPMV. These hydrogel depots were placed in the peritoneal cavity of mice, where they inhibited ovarian cancer cells and increased survival (Figure 9D–F) [116].

In summary, immunomodulatory and gene-based delivery systems are redefining ovarian cancer treatment by integrating chemotherapy, gene silencing, and immune activation into multifunctional nanomaterial systems. While targeted therapies demonstrate impressive specificity, the emerging chemodynamic and vaccine-based strategies offer something fundamentally different: the capacity to generate systemic immune responses that can attack even distant metastases. The convergence of stimuli-responsive release mechanisms (pH, MMP-2, GSH) with immune checkpoint inhibition (anti-PD-1) and innate immune activation (TLR agonists, bacterial adjuvants) suggests that next-generation therapies will succeed by orchestrating both immediate cytotoxicity and durable immunological memory.

#### 3.1.4. Localized, Controlled, and Advanced Formulation Systems

Ovarian cancer’s propensity for peritoneal dissemination demands depot formulations capable of sustained, site-specific drug exposure. Voznyuk developed a local chemotherapy platform for ovarian cancer treatment with biodegradable polymers. The platform features a polycaprolactone (PCL) core to ensure structural integrity and drug release in one direction. On the PCL core, a thin but multilayered coating (~200 nm) was deposited. This layer-by-layer coating comprised poly-γ-glutamic acid (γ-PGA) and either poly-l-lysine (PLL) or chitosan (CS). An ionic conjugate of γ-PGA and doxorubicin (DOX) was added to the multilayer coating to improve steady release. The quantity of empty (non-DOX) bilayers on this platform can be changed to control the drug release kinetics. In vitro, they showed anticancer action against ovarian cancer cells (SKOV3), while empty platforms (drugless) exhibited no cytotoxicity. These platforms can provide prolonged, steady drug release for more than 150 days [117]. A hydrogel by Diels–Alder reaction (reaction between furan-modified alginate and maleimido-PLGA-PEG triblock copolymer) was developed by Lim and colleagues, and it was loaded with oxaliplatin (OxPt) for controlled drug release. The Hydrogel-OxPt demonstrated an entrapment efficiency of 69.9 ± 0.9% and a controlled release profile, releasing 78.8% of oxaliplatin over a week, primarily via diffusion. Release mechanisms were confirmed by Korsmeyer–Peppas and Higuchi models with R^2^ values of 0.8924 and 0.9179, respectively [118]. Reovirus can target and kill ovarian cancer cells. However, ovarian cancer patients’ ascitic fluid contains antibodies that can neutralize reovirus. Yang et al. developed a cationic liposome that enhances the uptake of reovirus by caveolin-mediated endocytosis [119].

Vascular endothelial growth factor (VEGF) is an overexpressed protein in many gynecological cancers, including ovarian cancer. AB160, a 160 nm antibody-drug conjugate, was prepared by noncovalently binding bevacizumab to the albumin linkers present on the surface of nab-paclitaxel. Bevacizumab specifically targets VEGF. Recently, a phase I trial of AB160 showed promising results in the treatment of ovarian and other gynecological cancers [120]. Residual lesions that remain after tumor removal and unresectable lymph nodes with metastasis lead to poor survival among cancer patients. These issues are prominent in conditions like after the debulking surgery of ovarian cancer and retroperitoneal lymph node metastasis. Addressing these challenges requires local DDSs that offer continuous and stable delivery, high biocompatibility, and the capacity to deliver a variety of drugs. Zhang et al. created a drug-loaded hydrogel with precisely adjustable mesh size that is derived from pure DNA strands. This DNA hydrogel facilitates microcrystallization and subsequent sustained release of the radiosensitizing medication elimusertib. It also modulates the contact between immune cells and tumor cells. It provides a good strategy for improving local delivery, ensuring stable and steady treatment, and potentially synergizing with other therapies to enhance patient survival [121].

The recent use of poly [ADP-ribose] polymerase inhibitors (PARPi) in the treatment of ovarian malignancy has been credited with increased patient survival. However, oral administration of PARPi results in low bioavailability in cancer tissues and systemic toxicities, such as hematological disturbances, gastrointestinal upsets, and neurotoxicities. Therefore, there is a need for a localized DDS that can deliver PARPi successfully and safely. Injectable hydrogels can be utilized for these purposes. Han et al. prepared a hydrogel that can be injected using aminated hyaluronic acid and Pluronic 127 after aldehyde-functionalization through a Schiff base reaction. The obtained hydrogel, which loaded niraparib (a PARPi), demonstrated very good injectability, reduced time for gelation, and good self-healing properties. In vitro tests showed that the hydrogel (HP@Nir hydrogel) had a stable release profile and exhibited significant anti-proliferative as well as anti-migratory properties against OVCAR-3 cells. In vivo studies further confirmed the effectiveness of the HP@Nir hydrogel in suppressing tumor tissue growth without major side effects. This biocompatible hydrogel slowly degrades over approximately 20 days in vivo. It indicates that the injectable HP@Nir hydrogel can be an efficient strategy for treating ovarian cancer [122].

**Figure 10:**
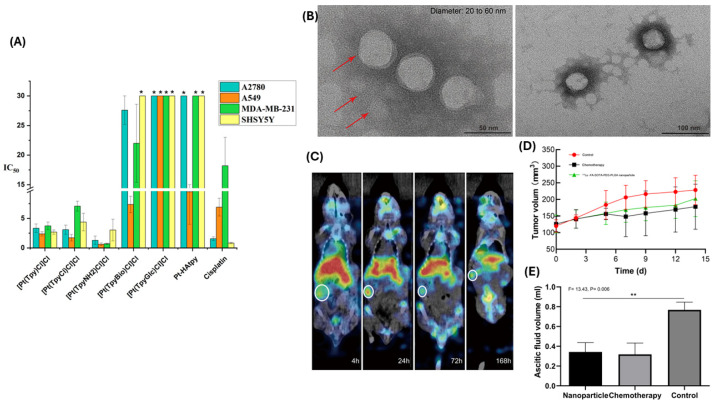
(**A**) IC_50_ values (in µM) of different platinum(II)–terpyridine complexes. * indicates an IC_50_ value > 30 µM. Reproduced from Panebianco et al., 2024, used under a Creative Commons CC BY 4.0 license [124]. (**B**) Nanoparticles prepared by Guo et al., 2024 [126], under transmission electron microscope at two different magnifications, (**C**) micro-single-photon emission computed tomography/computed tomography of tumor bearing mice after 4 h, 24 h, 72 h, and 168 h of injecting the nanoparticles, (**D**) tumor volumes plotted against time among different treatment groups, (**E**) volumes of ascitic fluid among different treatment groups. ** indicates *p* < 0.01. Reproduced from Guo et al., 2024, used under a Creative Commons CC BY 4.0 license [126].

Zeolitic imidazolate frameworks (ZIF) are a type of metal–organic framework (MOF) currently being explored for antitumor drug delivery. Entrapping carboplatin (CBP) in ZIF-90 nanoparticles, Chen et al. prepared a DDS for ovarian cancer. These nanoparticles were further modified with methoxypoly(ethylene glycol) having a folate (FA-PEG-NH_2_) on the aldehyde group of ZIF-90, forming CBP@ZIF-90-NPF nanoparticles. These NPs have an acid-responsive Schiff base bond. After 48 h, the authors observed that only 6.84% CBP was released at physiological pH, while 92.89% CBP was released in the acidic tumor microenvironment. Studies on cellular uptake revealed that FA-PEG-NH_2_ modified NPs exhibited particular selectivity for the nucleus of tumor cells. The CCK8 assay using malignant cells (OVCAR-3) and normal epithelial cells of the ovary (IOSE-80) showed good biocompatibility and minimal cytotoxicity for normal cells [123]. Panebianco et al. prepared platinum(II)- terpyridine complexes and modified the 4′ carbon with amine, glucose, biotin, and hyaluronic acid (one at a time). Then, they studied the cytotoxicity of the derived compounds against different cancer cell lines. They found that the platinum-terpyridine amino derivative compound was the most effective against ovarian adenocarcinoma cells (A2780), even outperforming cisplatin (Figure 10A) [124]. Nosrati et al. synthesized auraptene-nanostructured lipid carriers containing methylene blue. The nanocarriers underwent surface modification with folic acid-conjugated chitosan. During a cytotoxicity study against A2780 cells, it was observed that these nanoparticles upregulate apoptosis-inducing gene P53 and downregulate apoptosis-inhibiting genes Bax and Bcl-2 [125].

Guo et al. prepared nanoparticles from PLGA and PEG as the matrix. They modified these nanoparticles with folic acid to target folate receptors on ovarian cancer cells. They attached diethylene triamine pentaacetic acid to these nanoparticles as chelating agents and radiolabeled the nanoparticles with ^177^lutetium for local radiation effects. These nanoparticles were present in blood up to 72 h and exhibited low renal accumulation (1.646% ID/g). The therapeutic effects achieved with these nanoparticles were comparable to chemotherapy (Figure 10B–E) [126]. Varshosaz and colleagues prepared docetaxel-loaded electrosprayed nanoparticles and tested their effectiveness against the SKOV-3 mouse tumor model. They prepared an electrospraying solution by dissolving poly (butylene adipate-co-butylene terephthalate), PEG 6000, and docetaxel in a mixture of dichloromethane (DCM) and N,N-dimethylformamide (DMF). They determined that the optimum conditions for producing such nanoparticles are 20 kV, 12 cm of spinneret to collectorate distance, drug to polymer ratio of 1:3, and DCM to DMF ratio of 2.7:1 [127].

Song and colleagues synthesized an enantiomeric peptide nanoparticle by connecting a hexa-L-arginine segment and a lipid-like amphiphile with a disulfide bond. Suicidal gene and paclitaxel were loaded on these nanoparticles. Upon contact with GSH, the disulfide bond is cleaved, the nanoparticles take the shape of nanofibers, and the suicidal gene and paclitaxel are released. In vitro and in vivo studies confirmed the uptake of these nanoparticles by ovarian cancer cells and subsequent death [128]. Wen and colleagues copolymerized melphalan with p-dioxanone to convert it into a prodrug and shaped it into nanoparticles by electrospraying. These nanoparticles were phagocytosed by SKOV-3 cells, and lysosomal treatment freed melphalan from p-dioxanone. Electrosprayed nanoparticles showed better cytotoxicity against ovarian cancer cells than free melphalan, both in vitro and in vivo [129]. Similarly, coaxial fibers (core-polyvinyl alcohol and shell-chitosan) were electrospun for delivering doxorubicin to ovarian tumors [130].

The localized and advanced DDSs demonstrate the power of spatiotemporal control in ovarian cancer therapy. Across polymeric scaffolds, hydrogels, MOFs, and electrosprayed systems, the one shared goal is precision control over drug kinetics and microenvironmental interactions. The convergence of local radiotherapy, chemotherapy, and gene delivery within injectable or implantable systems signifies a shift toward personalized locoregional treatment. Discussions in Section 3.1.3 and Section 3.1.4 have been summarized in Table 3.

A particular challenge in advanced ovarian carcinoma is peritoneal metastasis with ascites. Large volumes of ascites can prevent reaching the effective concentration of the drug at the metastasis sites and accelerate net drug loss. This significantly shortens effective contact time with tumor nodules. Consequently, intraperitoneal approaches tend to be most effective against microscopic residual disease or small-volume tumor implants after maximal cytoreductive surgery and judicious ascitic fluid removal. Although hyperthermal intraperitoneal chemotherapy (HIPEC) as well as pressurized intraperitoneal aerosolized chemotherapy (PIPAC) are established methods of intraperitoneal drug delivery, these processes often result in peritoneal adhesion. Biomaterial-based drug delivery systems (esp. hydrogels) can help overcome these challenges by improving retention and providing a sustained release. With the end goal of intraperitoneal delivery, Surikutchi et al. prepared a covalently crosslinked PEG hydrogel and entrapped docetaxel-containing nanocapsules within the hydrogel. The nanocapsules remained in the intraperitoneal cavity for 24 h during the in vivo study (mouse). Within 24 h, the nanocapsules were completely released from the hydrogel and accumulated in the liver. The hydrogel itself degraded within a week [131]. Stimuli-responsive hydrogels can be injected as a liquid and then form a gel in the peritoneal cavity. Shin et al. have developed a hydrogel responsive to temperature for simultaneous intraperitoneal delivery of epothelione B, 17-AAG, and rapamycin. All the drugs were released within 12 h in mice [132]. Although hydrogel-based delivery systems can be effective in ascites, the main prerequisites are prolonged, sustained, and uniform release as well as timely degradation. Both of the works mentioned here have very short delivery times (24 h and 12 h, respectively).

### 3.2. Photothermal, Photodynamic, and Sonodynamic Therapies for Ovarian Cancer

In photothermal therapy, light is used to generate heat to kill cancer cells. Photodynamic therapy is a special type of photothermal therapy where light of a specific wavelength excites a photosensitizer, thus generating ROSs to kill cancer cells. The photosensitizer remains non-toxic until excited by the light source. Ye et al. developed nanoparticles for photodynamic therapy comprising folic acid, (triphenylamino-phenylaniline zinc phthalocyanine), and upconversion nanoparticles. They leveraged NIR laser-induced upconversion luminescence (UCL) for imaging and therapy. The emitted UCL enables real-time tracking within ovarian cancer cells (HO-8910). If activated by a laser of 980 nm, these nanoparticles generate singlet oxygens that exert cytotoxicity by disrupting the functions of mitochondria and inducing apoptosis. This showcases their potential for tumor ablation [133].

Despite recent advancements, the effectiveness of current immunogenic cell death (ICD) inducers is limited and often insufficient due to reliance on endoplasmic reticulum (ER) stress. Mitochondrial stress has shown higher efficacy in inducing large-scale ICDs. Zhang et al. introduced mitochondria-targeted stimuli-responsive polyprodrug nanoparticles (Mito-CMPN), which are superior ICD inducers. Mito-CMPNs were designed for chemo-photodynamic therapy, utilizing rhodamine B to target mitochondria, mitoxantrone (MTO) and cisplatin for synergistic immune-chemotherapy, and MTO for photodynamic immunotherapy, where it acts as a photosensitizer. Mito-CMPNs effectively reversed the immunosuppressive microenvironment in ovarian cancer models by inducing robust ICD through mitochondrial stress. The nanoparticles self-assembled from 2 polyprodrugs—P(MTO-TK) and P(OEGMA-co-RhBM)-b-PPtMA. Guided by the lipophilic RhB moiety, these nanoparticles could enter the mitochondria, where they generated ROS when irradiated by a laser of 660 nm wavelength, releasing damage-associated molecular patterns (DAMPs). As a result, the dendritic cells received signals to mature, and the tumor-associated macrophages polarized from M2 to M1-like phenotypes, activating both innate and adaptive immunity. In vivo studies demonstrated delayed progression of tumor tissue and reduced metastasis [134]. These photodynamic approaches reveal a critical design principle: targeting specific organelles dramatically enhances therapeutic outcomes. While Ye et al.’s upconversion nanoparticles demonstrated proof-of-concept for imaging-guided therapy, Zhang et al.’s mitochondria-targeting strategy achieved superior immunogenic effects by shifting the stress mechanism from the endoplasmic reticulum to mitochondria. The key advancement lies not merely in ROS generation, but in strategically directing photosensitizers to subcellular compartments where oxidative damage can trigger the most robust antitumor immune responses.

**Figure 11:**
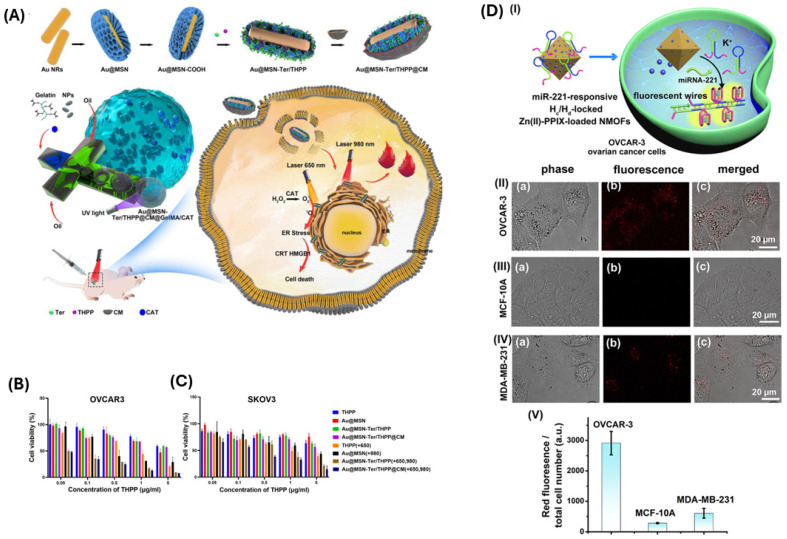
(**A**) Schematic showing the preparation and mechanism of action of the nanoparticles prepared by Ma et al., 2023 [137], (**B**) viability of OVACAR3, and (**C**) SKOV3 cells after co-incubation with different concentrations of different nanoparticle formulations and irradiation with 650 nm and/or 980 nm lasers for 24 h. Reproduced from Ma et al., 2023, used under a Creative Commons CC BY 4.0 license [137]. (**D**(**I**)) Schematic showing the mechanism of action of miR-221-responsive NMOF nanoparticles. (**D**(**II**–**IV**)) Confocal microscopy images of OVCAR-3, MCF-10A, and MDA-MB-231 cells, respectively. (**D**(**V**)) Integrated normalized fluorescence intensities of the mentioned 3 types of cells after treatment with NMOF. Reproduced from Zhang et al., 2022, used under a Creative Commons CC-BY 4.0 license [139].

Fan and coworkers prepared nanostructured lipid carriers loaded with camptothecin (CPT) and gold nanocomposites (AuNCs) for dual chemo/photothermal therapy. Gold nanorods (AuNRs) and nanospheres (AuNSs) were incorporated to utilize their photothermal properties. Upon near-infrared (808 nm) exposure, AuNRs showed the highest heating potential (ΔT = 22 °C in 5 min) and enhanced CPT release. The CPT + AuNRs@NSLCs formulation demonstrated significant cytotoxicity, inhibiting 81% of HeLa ovarian cancer cell growth under 5 min of irradiation. One limitation of this preparation is poor loading of the drug (9.12%) [135]. Carbon-coated MoSe_2_ (MEC) nanoparticles were synthesized by a single-step hydrothermal method by Yu et al. Biological studies demonstrated that MEC combined with NIR laser irradiation effectively reduced SKOV-3 ovarian cancer cell viability to 61.6% ± 8.9%. Photothermal therapy using MEC nanoparticles showed significant tumor suppression in mice without adverse effects on other organs. MEC’s high absorbance, as well as heat generation under laser irradiation, contributed to the enhanced therapeutic outcomes [136]. Ma and colleagues prepared gold nanorod-mesoporous silica core–shell nanoparticles. They loaded a photosensitizer, porphine, into these nanoparticles. Finally, these nanoparticles were seeded with catalase into a gelatin methacryloyl microgel matrix to enhance photodynamic reaction and photothermal conversion. These nanoparticles were further modified with Tosyl Ethylenediamine for targeting the endoplasmic reticulum. These nanoparticles caused prolonged stress in the endoplasmic reticulum mediated by photodynamic reactions and resulted in immunogenic death of ovarian cancer cells (Figure 11A–C) [137].

Pheophorbide A generates ROS when stimulated by NIR. Lee et al. prepared hyaluronan-cholesterol nanoparticles. They attached SN38 (an anticancer compound) to these nanoparticles using a thioketal (TK) linker. This TK linker is cleaved by ROS. Upon application of NIR, a synergistic action of chemotherapy (SN38) and photodynamic therapy caused the death of invasive HEY-T30 ovarian cancer cells [138]. Zhang and coworkers have developed a platform for photodynamic therapy and miRNA-mediated fluorescence imaging of cancer cells based on UiO-66-NH_2_ nano metal–organic framework (NMOFs). Zn(II)-protophyrin IX was loaded on these NMOFs, and the NMOFs were gated with DNA hairpins containing miRNA recognition sequences (Figure 11D) [139].

**Figure 12:**
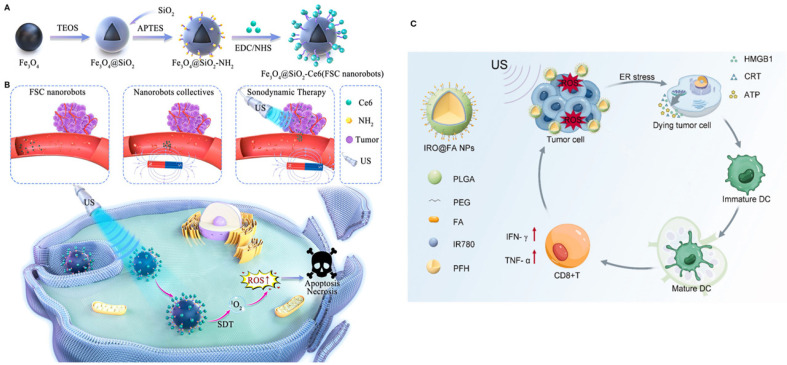
(**A**,**B**) Schematic of the preparation and mechanism of action of sonodynamic nanorobots. Reproduced from Zhou et al., 2024, used under a Creative Commons CC BY 4.0 license [140]. (**C**) Mechanism of immunomodulation by sonosensitizer nanoparticles. Reproduced from Jing Zheng et al., 2022, used under a Creative Commons CC BY license [144].

In sonodynamic therapy, a chemical sonosensitizer is activated at the target site with low-dose ultrasound to produce ROS. Zhou et al. coated Fe_3_O_4_ nanoparticles with SiO_2_ and attached a sonosensitizer, chlorin e6 (Ce6), to these nanoparticles to create sonodynamic nanorobots. Under the applied magnetic field, these nanorobots accumulated in the target site where they were later activated using low-intensity ultrasound to kill ovarian cancer cells (ID8 cells) (Figure 12A,B) [140]. Hypoxic tumor microenvironment (TME) limits ROS production and thereby limits the efficacy of sonodynamic therapy. Platinum-boron-phosphorus ternary nanoparticles with Ce6 were synthesized by Yue et al. to enhance the catalytic decomposition of H_2_O_2_ in hypoxic TME. The authors tested these nanoparticles against SKOV3 ovarian cancer cells. These ternary nanoparticles can function with the same efficacy at pH as low as 1, up to 50 °C temperature, high-intensity ultrasound (2W), and after a storage time of 1 month. They also showed less toxicity when compared to platinum nanoparticles [141]. Lee et al. used 4-arm polyethylene glycol (PEG) to functionalize graphene nanoribbons (GNR) and attached Ce6 to GNRs to target metastatic ovarian cancer spheroids. GNR-PEG-Ce6 disrupts the adhesion of the spheroids to extracellular matrix proteins and LP-9 mesothelial cells. Combined with mild ultrasound irradiation, Ce6 induces sonodynamic effects that kill adhered spheroids. GNR-PEG-Ce6 also loosens cell–cell adhesions. This enhances their susceptibility to chemotherapeutic agents like cisplatin and paclitaxel [142]. Sonodynamic therapy emerged as an alternative to photodynamic approaches, particularly for addressing tumor hypoxia and metastatic disease. While Zhou et al.’s magnetic guidance system enables precise nanorobot positioning, Yue et al.’s platinum-boron-phosphorus nanoparticles tackle the more fundamental problem of hypoxia-limited ROS generation in cancer tissues. Lee et al.’s graphene nanoribbon system reveals that sonodynamic effects extend beyond direct cytotoxicity to mechanically disrupting spheroid architecture, thereby sensitizing metastatic clusters to conventional chemotherapy. The clinical advantage of sonodynamic therapy over photodynamic approaches lies in ultrasound’s superior tissue penetration. This property makes sonodynamic therapy particularly promising for treating deep-seated and disseminated ovarian cancer.

Jiao Zheng et al. have prepared a core (perfluoropentane)-shell (PLGA) nanoparticle that can be utilized for delivering chemotherapy and photo-sonodynamic therapy. The core contains oxygen, and the shell contains oxaliplatin and a photosensitizer, indocyanine green. They can also be utilized as contrast agents for photoacoustic imaging, thereby enabling their tracking. They are also capable of inducing immunogenic death [143]. Jing Zheng et al [144]. prepared nanoparticles from PLGA and PEG for sonodynamic therapy in ovarian cancer and modified the nanoparticles with folic acid. They used IR780 as the sonosensitizer and perfluorohexane as the oxygen-carrying substance (Figure 12C) [144]. Human serum albumin (HSA) was loaded with Ce6 and catalase to synthesize nanoparticles by Zhang et al [144]. They later attached HER-2 nanobody to these nanoparticles via a heterodisulfide bond. Under 660 nm NIR laser irradiation, these nanoparticles generated abundant ROS. They induced apoptosis in SKOV-3 cells via ROS and anti-CTLA-4 activities [145]. Pinto and coworkers encapsulated meta(tetrahydroxyphenyl)chlorin, a 2nd generation photosensitizer, inside extracellular vesicles harvested from mesenchymal stem cells for enhanced photodynamic therapy against peritoneal metastasis [146]. With the same aim to target peritoneal metastasis, Luan and colleagues encapsulated methyl aminolevulinate, which is a photosensitizer within liposomes (lipMAL), adopting the ammonium sulfate gradient method. These liposomes showed superior performance in photodynamic diagnosis and photodynamic therapy against SKOV3 cells compared to free MAL [147]. IR780 iodide can be utilized in both photothermal and photodynamic therapy. However, the limitations of this agent are hydrophobicity and toxicity. To overcome these issues, Potara et al. encapsulated IR780 within amphiphilic pluronic-F127-chitosan nanocapsules modified with folic acid (IR780-chit-FA). These nanocapsules were successfully internalized within NIH-OVCAR-3 cells, and dual photodynamic-photothermal therapy was achieved after irradiation at 785 nm for 5 min [148]. The aforementioned combination therapies can be utilized to achieve better efficacy through their synergistic effects than single-therapy approaches.

## 4. Current Status, Challenges, and Future Perspectives

Currently, the number of approved biomaterial-based therapies for ovarian cancer is very limited. PEGylated liposomal doxorubicin (Doxil^®^/Caelyx^®^) was approved in 1999 for platinum-refractory ovarian cancer. In 2005, it gained full approval for platinum-sensitive recurrent disease. It remains a key option for treating recurrence in ovarian cancer, both as monotherapy and in combination regimens. The liposomal formulation prolongs the circulation time of doxorubicin and exploits the enhanced permeability and retention effect in tumor tissue to increase drug delivery. Doxorubicin causes cardiotoxicity. Liposomal encapsulation reduces cardiac toxicity [149]. Genexol-PM is a formulation without Cremophor EL where paclitaxel is loaded in polymeric micelles. Two phase I trials in ovarian cancer patients—one in the United States (maximum tolerated dose-MTD 435 mg/m^2^) and one in Korea (MTD 390 mg/m^2^)—established a recommended dose of 300 mg/m^2^ at three-week intervals. A randomized phase II trial (NCT01276548) compared Genexol-PM + carboplatin versus Cremophor EL-based paclitaxel + carboplatin. Genexol-PM showed non-inferior efficacy (overall response rate 88% vs. 77.1%) and a similar safety profile [150,151].

The FDA approved albumin-bound paclitaxel (nab-paclitaxel; Abraxane^®^) for treatment-resistant metastatic breast cancer, metastatic adenocarcinoma of the pancreas, and locally advanced or metastatic non-small cell lung cancer [FDA label of ABRAXANE]. It has been evaluated in the recurrence of ovarian cancer, where the cancer remains platinum-sensitive. In a one-agent phase II trial, 44 patients received 260 mg/m^2^ of Abraxane^®^ every 21 days for 6 cycles or until the disease progressed. The overall response rate was 64%. Median progression-free survival was 8.5 months. Serious side effects were relatively less. 11% of patients experienced grade 4 neutropenia, and 13% suffered from grade 2–3 neuropathy. There was no hypersensitivity reaction. Combination trials of nab-paclitaxel with carboplatin also reported successful antitumor activity as well as acceptable tolerability [152,153]. Recently (November 2022), mirvetuximab soravtansine-gynx (MIRV) received full FDA clearance for the treatment of several folate receptor α-positive gynecological cancers. It belongs to the antibody-drug conjugate (ADC) class and consists of a monoclonal antibody against FRα and a microtubule inhibitor. Another FDA-approved ADC is tisotumab vedotin for metastatic or recurrent cervical cancer. Here, a tissue factor targeting monoclonal antibody is conjugated with a microtubule inhibitor. ADCs are prescribed after standard treatment options are exhausted. Currently, several other ADCs for ovarian cancer targeting FRα, TROP-2, MSLN, HER2, NaPi2b, DPEP3, and tissue factor are being investigated [154]. CRLX101 is a cyclodextrin-polymer–camptothecin conjugate that self-assembles into approximately 30–40 nm nanoparticles. A phase 1/2a trial in advanced solid tumors (including ovarian cancer) determined a safe biweekly dose of 15 mg/m^2^ for CRLX101. A subsequent phase II study is evaluating CRLX101, with or without bevacizumab, in patients with fallopian tube, ovarian, or primary peritoneal cancer (NCI-2012-01544) [155]. clinicaltrials.gov was searched with the same keywords used for retrieving the articles for this literature review. The time span chosen was from 2020 to the current day. A total of 74 articles were returned in the search result. Most of the trials completed or initiated in the last 5 years are liposome-based. If liposome-based trials are excluded, only 6 clinical trials remain (Table 4), which utilize different types of drug nanoparticles.

Despite the promises of biomaterials in the diagnosis, screening, and treatment of ovarian cancer, several significant challenges remain–chief among them being biocompatibility and toxicity. The translation of biomaterial-based platforms in clinical settings demands rigorous evaluation of their interaction with biological systems to ensure safety, efficacy, and minimal adverse effects.

The ability of a material to carry out its intended function within a living system, causing no or minimal toxicity, injury, or adverse immune or physiological responses, is known as biocompatibility [156]. While many synthetic polymers (e.g., PLGA, PEG) and natural materials (e.g., chitosan, alginate) have demonstrated favorable biocompatibility profiles in preclinical studies, their behavior in complex human physiological environments can be unpredictable. For instance, nanoparticle-based drug delivery systems may inadvertently activate immune responses or accumulate in off-target tissues, leading to inflammation or organ toxicity [157]. Studies have also shown that surface charge, size, shape, and chemical nature of biomaterials significantly influence uptake by cells, biodistribution, and clearance mechanisms, which in turn affect their safety profiles [158,159].

Another significant worry is the toxicity of certain biomaterials in the long term, particularly those that are non-biodegradable or have degradation by-products that may accumulate over time. For example, some metal-based nanoparticles (e.g., gold, iron oxide) have shown persistence in tissues, raising the possibility of chronic toxicity and disturbances in normal cellular processes [160]. Therefore, understanding the degradation kinetics and metabolic fate of biomaterials is critical before advancing to human trials.

Developing scalable, Good Manufacturing Practices (GMP)-compliant manufacturing processes remains another valid concern. Critical translational bottlenecks include achieving batch-to-batch reproducibility. This is a big challenge, particularly for complex formulations involving multiple components such as polymers, drugs, and targeting ligands. Scale-up from laboratory to industrial production is often met with unforeseen challenges. In many cases, commercial translation of successful lab results is not economically feasible. Additionally, ensuring sterility and endotoxin control becomes increasingly difficult as production scales increase. Sophisticated aseptic processing techniques and comprehensive quality control measures are required to ensure sterility. Good Laboratory Practice (GLP) compliance during preclinical studies and Chemistry, Manufacturing, and Controls (CMC) documentation for regulatory submissions demand substantial resources and expertise that many academic laboratories and early-stage companies lack.

Regulatory considerations further complicate clinical translation. Regulatory agencies like the European Medicines Agency or the U.S. Food and Drug Administration require extensive in vitro and in vivo toxicity data, pharmacokinetics, and efficacy profiles before approving biomaterial-based products. The regulatory pathway becomes particularly complex for biomaterial-based platforms that may be classified as combination products (drug-device combinations). These combination products require coordination between different regulatory divisions and potentially multiple approval pathways. The absence of established protocols for evaluating complex biomaterial systems (such as hybrid platforms combining drugs, targeting ligands, and polymers) poses a challenge to streamlined approval processes [161]. Furthermore, the absence of long-term clinical data for many biomaterial systems limits their adoption in oncology, including ovarian cancer treatment.

To bridge this gap, interdisciplinary collaboration is essential. Materials scientists, oncologists, pharmacologists, and regulatory experts must work together to design biomaterials that are not only effective but also safe and compliant with clinical standards. In silico modeling, organ-on-chip platforms, and advanced in vivo imaging techniques offer powerful tools to predict and monitor the behavior of biomaterials in real-time, potentially reducing reliance on traditional animal models and accelerating clinical translation. In this regard, artificial intelligence can accelerate nanocarrier design by predicting optimal size, charge, and surface chemistry. Machine learning can guide the selection of targeting ligands tailored to each patient. Biomaterial-based sensors will be feeding real-time data into precision-medicine platforms. Ultimately, implants or nanoparticles may be individualized to a patient’s genetic profile and tumor biomarker signature.

## 5. Conclusions

In conclusion, while biomaterials hold immense potential to transform ovarian cancer management, addressing safety concerns, ensuring biocompatibility, and complying with regulatory frameworks remain essential for their successful clinical applications. Ongoing efforts to develop standardized testing frameworks and next-generation, patient-specific biomaterials will ultimately lead to safer and more successful ovarian cancer therapies.

## Figures and Tables

**Table 2:** Biomaterial-based targeted, stimuli-responsive, and combination therapies for ovarian cancer.

Delivery System	Drug(s)	Targeting Mechanism	Stimuli/Trigger	Key Findings	Reference
Octreotide-modified liposome	Paclitaxel, Neferine	SSTR2-mediated endocytosis	-	Enhanced targeting via somatostatin receptor binding; extended circulation time	[88]
Dual-functionalized mesoporous silica nanobean	Doxorubicin	Lysosomal/mitochondrial localization	Elevated GSH levels, reduced pH	Disulfide bond cleavage activates fluorescence and drug release; mitochondrial dysfunction and autophagy induction	[89]
Thermoresponsive polymer nanoparticles made from (p(SB-co-ZB)) copolymers and vinyl oligoesters	Paclitaxel	Temperature-triggered release	Hyperthermia (43 °C)	Nearly complete drug release at 43 °C while retaining >95% at physiological temperature (37 °C)	[90]
Supramolecular organic framework	Doxorubicin	cRGD peptide targeting	Binding with lysophosphatidic acid (LPA)	SOF disassembly and LPA binding; combined chemotherapy and photodynamic therapy	[91]
ZHER2:342-Epo B Affibody-Drug Conjugate Nanoagent	Epothilone B	HER2 receptor targeting	Elevated reactive oxygen species	ROS-sensitive thioketal group enables drug release at the target site	[92]
Methacryloyl gelatin microneedle	Ginsenoside RG3	Direct tumor penetration	Mechanical insertion	Bypasses first-pass metabolism; optimal release kinetics with enhanced bioavailability	[93]
Magnetic mesoporous silica nanoparticle	Cisplatin	External magnetic field guidance	Acidic tumor environment	Deep tumor penetration; pH-responsive drug release with reduced systemic toxicity	[94]
pH-responsive polymeric micelle	Doxorubicin, Paclitaxel, Gossypol, SN38	pH-responsive release	pH changes (<6.5)	Rapid drug release triggered by acidic pH; enhanced cytotoxicity compared to non-responsive systems	[95]
Trastuzumab-modified liposome	Oxaliplatin	HER2 receptor targeting	Peptide linker cleavage by Matrix metalloproteinases	PEG layer removal leads to liposome destabilization and drug release	[96]
Ephrin-B2 and LAMP2b modified extracellular vesicle (EV)	-	Ephrin-B4 receptor targeting	Cellular internalization	Enhanced EV internalization compared to unmodified vesicles in vitro and in vivo	[97]
Tetramethylpyrazine-loaded exosome	Paclitaxel	Natural EV-mediated delivery	Cellular uptake	Overcame drug resistance by downregulating MDR proteins; enhanced apoptosis in resistant cells	[98]
PLGA nanoparticle	PF543 + Carboplatin	SphK1 inhibition	Targets pro-survival pathways regulated by sphingosine kinase 1	Sensitized platinum-resistant SKOV3 cells to carboplatin in vitro and in vivo	[99]
TPGS-Soluplus^®^ nanomicelle	Albendazole + Paclitaxel	Cellular entry via folic acid receptor	Dual drug delivery with sustained release profile	Superior cytotoxicity compared with free drugs; effective tumor targeting and penetration over 90 h	[100]
Folic acid receptor-targeted nanoparticle	Cisplatin	BACH1/CD47 inhibition	Inhibits BACH1 and CD47, promotes apoptosis, enhances macrophage phagocytosis	Increased M1 macrophages in tumors; potential for platinum-resistant ovarian cancer	[101]
PLGA-PEG nanoparticle	Paclitaxel + Atovaquone + Quercetin	ATP depletion	Inhibits oxidative phosphorylation and glycolysis to deplete ATP	Suppressed P-gp activity, increased drug accumulation, halted tumor growth in in vivo A2780/Taxol model	[102]
PEG-thioketal-hyaluronic acid- paclitaxel and diosgenin liposome	Paclitaxel + Diosgenin	CD44 targeting via hyaluronic acid	Long circulation time with targeted accumulation	Prolonged circulation time with enhanced drug delivery to tumor site	[103]
Nucleic acid aptamer-modified zinc ferrite nanoparticle	Zinc ferrite + Cisplatin	Tumor-targeting aptamer (NucA)	Induces autophagy via zinc ion release and ROS generation	Enhanced cisplatin sensitivity; dual capabilities (therapeutic and MRI contrast)	[104]
Ferroplatinum nanoliposome	Cisplatin	Folic acid receptor targeting	Dual therapeutic and diagnostic approach	Combined chemotherapy with imaging capabilities	[105]
Fe_3_O_4_-porous organic cage CC3 nanocomposite	Fe_3_O_4_ + CC3 cage	Direct tumor targeting	CytC/caspase-3 apoptotic pathway activation	Significant inhibition of SKOV3 subcutaneous tumors in nude mice	[106]
chitosan oligosaccharide—d-alpha-tocopheryl polyethylene glycol 1000 succinate micelle	Bufalin	RGD peptide-mediated targeting	Targets integrin αvβ3 receptors	Reduced migration/invasion of cancerous cells, induction of apoptosis, decreased tumor burden in intraperitoneal metastasis model	[107]

**Table 3:** Biomaterial-based immunotherapies, gene therapies, and other advanced drug delivery systems.

Delivery System	Therapeutic Agent	Mechanism/Target	Key Findings	Reference
Cell membrane-camouflaged nanoparticle	Mitoxantrone, Her2 antisense oligonucleotides	RBC membrane for immune evasion, cancer cell membrane for targeting	75.7% apoptosis in vitro, 83.3% tumor suppression in vivo	[108]
DNA Tubular Origami	Fluorescent-labeled DNA strands	miR-21 downregulation	Significant decrease in miR-21 expression; effective cargo protection	[109]
Lipid nanoparticle	HER2-CD3-Fc bispecific antibody	HER2 and CD3 dual targeting caused T-cell activation	Strong T cell-directed cytotoxicity against HER2-positive cells	[110]
FeS@MR-1 bacterial nanoparticle	FeS present on *Shewanella oneidensis* MR-1	Chemodynamic therapy via Fenton reaction	H_2_S and Fe(II) release; systemic antitumor responses	[111]
Extracellular vesicle mimetic nanovesicle	β-Lapachone, iron oxide nanoparticles	•OH generation and GSH depletion	H_2_O_2_ generation and enhanced oxidative stress via GSH depletion	[112]
Injectable nanohydrogel	Paclitaxel, anti-PD-1 antibody	Sustained release of chemotherapy and immunotherapy agents from hydrogel	Sustained release over 7 days; suppression of metastasis	[113]
EGFR-targeted mesoporous silica nanoparticle	Doxorubicin	EGFR targeting with husA modification of mesoporous silica nanoparticles	Very high drug-to-antibody ratio (11.8) for an antibody-drug conjugate; enzyme/pH-responsive release	[114]
Smart nanogel vaccine system	CpG@Man-P, trametinib, PD-1 antibody	TLR9 activation and tumor antigen generation	40 nm particles with superior lymph node accumulation, tumor growth and metastasis suppression	[115]
CPMV-loaded hydrogel depot	Cowpea mosaic virus	TLR agonist (TLR 2, 4, 7) and innate immune cell activation	Sustained immunogenicity; improved survival with slow-release depots	[116]
Biodegradable polymer platform	Doxorubicin	PCL core with multilayer coating of γ-PGA	Controlled release > 150 days	[117]
Diels-Alder hydrogel	Oxaliplatin	Crosslinking between furan-modified alginate and maleimido-PLGA-PEG triblock copolymer	69.9% entrapment efficiency; 78.8% release over one week	[118]
Cationic liposome	Reovirus	Caveolin-mediated endocytosis	Enhanced reovirus uptake despite the presence of neutralizing antibodies	[119]
AB160 antibody-drug conjugate	Paclitaxel	Bevacizumab-paclitaxel noncovalent binding	Phase I trial has been completed	[120]
DNA hydrogel	Elimusertib	DNA strand-derived hydrogel for drug delivery	Microcrystallization of radiosensitizer elimusertib and its sustained release	[121]
Injectable hydrogel	Niraparib	Hydrogel made from hyaluronic acid and pluronic127	Good injectability; 20-day biodegradation; anti-proliferative and anti-migratory effects	[122]
ZIF-90 nanoparticle	Carboplatin	Folate-PEG-NH_2_ modification with acid-responsive Schiff base bond	6.84% release at physiological pH; 92.89% in acidic tumor environment	[123]
Platinum(II)-terpyridine complex	Platinum derivatives	4′ carbon modification with amine, glucose, biotin, and hyaluronic acid (one at a time)	Platinum-terpyridine amino derivative compound outperformed cisplatin in cytotoxicity	[124]
Nanostructured lipid carrier	Auraptene, methylene blue	Folic acid-conjugated chitosan surface modification	P53 upregulation; Bax and Bcl-2 downregulation	[125]
Radiolabeled PLGA-PEG nanoparticle	^177^Lutetium	Folic acid receptor targeting	72 h blood circulation; low renal accumulation; comparable to chemotherapy	[126]
Electrosprayed nanoparticle	Docetaxel	Nanoparticles prepared from poly(butylene adipate-co-terephthalate)	Optimized conditions: 20 kV, 12 cm distance, 1:3 drug:polymer ratio	[127]
Enantiomeric peptide nanoparticle	Suicidal gene, paclitaxel	GSH-responsive disulfide bond cleavage	Transformation from particle to fiber upon GSH contact; dual therapeutic release	[128]
Melphalan prodrug nanoparticle	Melphalan-p-dioxanone copolymer	Lysosomal activation of electrosprayed prodrug	Enhanced cytotoxicity compared to free melphalan	[129]
Coaxial electrospun fiber	Doxorubicin	Core-PVA, shell-chitosan	Controlled drug delivery from electrospun fibers	[130]

**Table 4:** Clinical trials on nanosystems other than liposomes.

National Clinical Trial (NCT) Number	Study Title	Study Status	Interventions
NCT04778839	Study of Paclitaxel Micelles for Injection in Chinese Patients With Advanced Solid Tumors.	RECRUITING	Drug: Paclitaxel Micelles for Injection
NCT04669002	EP0057 in Combination With Olaparib in Advanced Ovarian Cancer	COMPLETED	Drug: EP0057 (cyclodextrin-camptothecin nanoparticles) Drug: Olaparib tablets
NCT06048367	Carbon Nanoparticle-Loaded Iron [CNSI-Fe(II)] in the Treatment of Advanced Solid Tumor	COMPLETED	Drug: CNSI-Fe(II) nanoparticles 30/60/90/120/150 mg
NCT05001282	A Study to Evaluate ELU001 in Patients With Solid Tumors That Overexpress Folate Receptor Alpha (FRÎ±)	TERMINATED	Drug: ELU001 nanoparticles (consist of ~13 folic acid targeting moieties and a payload of ~22 molecules of the topoisomerase-1 inhibitor, exatecan)
NCT05969041	Study of MT-302 in Adults With Advanced or Metastatic Epithelial Tumors	RECRUITING	Drug: MT-302 (TROP2-targeting mRNA-based CAR therapy)
NCT05092373	Phase I Study of Tumor Treating Fields (TTF) in Combination With Cabozantinib or With Pembrolizumab and Nab-Paclitaxel in Patients With Advanced Solid Tumors Involving the Abdomen or Thorax	RECRUITING	Biological: Atezolizumab Drug: Cabozantinib S-malate Drug: Nab-paclitaxel

## Data Availability

No new data were created or analyzed in this study.
